# A Systematic Review of Ebola Treatment Trials to Assess the Extent to Which They Adhere to Ethical Guidelines

**DOI:** 10.1371/journal.pone.0168975

**Published:** 2017-01-17

**Authors:** Thomas Richardson, Andrew McDonald Johnston, Heather Draper

**Affiliations:** 1 College of Medical and Dental Sciences, University of Birmingham, Birmingham, United Kingdom; 2 Academic Department of Military Anaesthesia and Critical Care, Royal Centre for Defence Medicine, Queen Elizabeth Hospital, Birmingham, United Kingdom; 3 Institute for Applied Health Research, University of Birmingham, Birmingham, United Kingdom; University of Liverpool, UNITED KINGDOM

## Abstract

**Background:**

**Objective:** To determine to what extent each trial met criteria specified in three research frameworks for ethical trial conduct.

**Design:** Systematic review and narrative analysis

**Methods and findings:**

**Data sources:** MEDBASE and EMBASE databases were searched using a specific search strategy. The Cochrane database for systematic reviews, the PROSPERO database and trial registries were examined. A grey literature search and citation search were also carried out.

**Eligibility criteria for selecting studies:** Studies were included where the intervention was being used to treat Ebola in human subjects regardless of study design, comparator or outcome measured. Studies were eligible if they had taken place after the 21^st^ March 2014. Unpublished as well as published studies were included.

**Included studies:** Sixteen studies were included in the data synthesis. Data was extracted on study characteristics as well as any information relating to ten ethical areas of interest specified in the three research frameworks for ethical trial conduct and an additional criterion of whether the study received ethics approval from a research ethics committee.

**Synthesis of results:** Eight studies were judged to fully comply with all eleven criteria. The other eight studies all had at least one criteria where there was not enough information available to draw any conclusions. In two studies there were ethical concerns regarding the information provided in relation to at least one ethical criteria.

**Description of the effect:** One study did not receive ethical approval as the authors argued that treating approximately one hundred patients consecutively for compassionate reasons did not constitute a clinical trial. Furthermore, after the patients were treated, physicians in Sierra Leone did not release reports of treatment results and so study conclusions had to be made based on unpublished observations. In another study the risk-benefit ratio of the trial drug does not appear to be favourable and the pre-trial evidence base for its effectiveness against Ebola is speculative.

**Conclusions:**

Some limited and appropriate deviation from standard research expectations in disaster situations is increasingly accepted. However, this is not an excuse for poor ethics oversight and international regulations are in place which should not be ignored. New guidelines are needed that better define the boundaries between using medicines for compassionate use and conducting a clinical trial. Greater support should be offered for local research ethics committees in affected areas so that they can provide robust ethical review. Further systematic reviews should be carried out in epidemics of any novel infectious diseases to assess if comparable findings arise.

## Introduction

### Background

The 2014 Ebola outbreak is the most widespread epidemic of the highly lethal viral haemorrhagic disease with 11, 310 confirmed deaths as of the 10^th^ June 2016 [1]. It resulted in a rapid increase in research into potential Ebola treatments and vaccines [2,3]. However, as of November 2016, there is no licensed drug therapy against Ebola and currently recovery is dependent on a combination of best supportive care and the patient’s immune response [4].

It is argued that deviation from standard research expectations to an appropriate extent in a disaster setting may be acceptable [5–7]. In the case of Ebola, in August 2014, the World Health Organisation (WHO) concluded that it would be acceptable to use unregistered treatments that had shown promising results in animal models but had not been tested for safety or efficacy in humans [8]. Importantly, this was conditional on certain criteria being met, including a number of ethical requirements. These include but are not limited to: transparency about care, fairness, informed consent, freedom of choice, confidentiality, respect for the person, preserving dignity, risk-benefit assessment and community involvement [8].

#### Why is this review important?

According to the most recent WHO report on research and development of Ebola therapies, there are fifteen Ebola treatment trials currently in progress [9]. Questions have been raised about whether two of these were carried out in an ethically appropriate manner, relative to exceptional circumstances of the Ebola crisis:

1. Fedson et al. prescribed approximately 100 consecutive patients atorvastatin and irbesartan under compassionate use to treat Ebola [10]. The authors stated a 2% mortality rate was achieved, compared to a mean mortality of 40% in the current outbreak [1]. However, there was no formal documentation of the patients’ treatment and these mortality rates appear to be given verbally as an unpublished observation by one of the study authors [10].
2. One study was registered on the Pan African Clinical Trials Registry Committee but was later withdrawn [11]. It proposed assessing the efficacy and safety of amiodarone in Ebola patients despite pre-clinical data on antiviral activity of amiodarone being extremely limited [12]. Previously, amiodarone had been given as a compassionate therapy to 65 patients with Ebola, of whom 63% died. The authors acknowledge that there was no evidence to suggest that amiodarone had any beneficial effect on these patients [12]. Therefore, some clinicians have asked whether, given amiodarone’s potentially toxic side-effects, it is ethically acceptable to give amiodarone off-licence to treat Ebola [13].

A systematic review of all Ebola treatment trials to determine the extent to which studies have complied with international and local ethical standards for research is therefore timely. If trials have not conformed to ethical norms, then this may suggest a greater need for transparency and accountability from researchers in any future situation where a novel infectious disease precipitates a medical emergency; such as a new strain of influenza.

### Objectives

The aim of this systematic review is to examine all Ebola treatment trials that were conducted during the 2014 outbreak and systematically review whether they satisfied international research ethics requirements. Three published ethical frameworks were used to derive a set of criteria against which to compare studies. These were those published by the WHO, Médecins Sans Frontières (MSF) and a framework by Emanuel, Wendler and Grady which has been designed specifically for use in developing countries [14–16].

These frameworks were initially chosen as they are peer-reviewed, internationally recognised frameworks. In a recent literature review, the Research for Health in Humanitarian Crises described all three ethical frameworks as milestones in addressing ethical concerns in disaster settings [17]. Secondly, the frameworks were developed by organisations from differing backgrounds- a specialised agency within the United Nations (WHO), a global non-governmental organisation (MSF) and a research team from the National Institutes of Health (Emanuel et al.). Therefore, the frameworks contain subtle differences accommodating the wide range of opinions about the essential ethical components for ethical trial conduct. These differences will be compared in more detail later in this review.

The objectives of this systematic review are:

#### Primary Objective

To determine the extent to which each trial meet criteria specified in the three research frameworks for ethical trial conduct.

#### Secondary Objective

To compare the three ethical frameworks and to suggest how they can be modified and improved in light of the systematic review.

## Methods

### Protocol and registration

The systematic review protocol was ineligible for registration on the PROSPERO database because it is concerned solely with the ethics of clinical trials and, as such, has no directly health-related outcomes. The protocol for this systematic review is reported in S3 Appendix.

### Eligibility criteria

#### Study characteristics

Study design: The review included all study designs. Studies were eligible for inclusion even if the trial had been withdrawn as this reflects the ethical implications of starting a trial and not completing it.

Populations: Human subjects of any age who are suspected to have Ebola.

Interventions: Any intervention to treat Ebola. Vaccine studies were excluded as this would broaden the scope of the review too much given the time constraints of the author’s degree program.

Comparators: All comparators were eligible for inclusion.

Outcomes: Trials were included regardless of what outcomes they reported as these need to be assessed to see whether they are ethically acceptable.

Timing: Studies were eligible if they took place after the Ebola outbreak was first identified on the 21^st^ March 2014 [18].

Setting: No restrictions on the location of trials.

#### Report characteristics

Language: Non-English studies were eligible for inclusion.

Publication status: Unpublished as well as published articles were identified as unpublished trials may have been less likely to satisfy ethical criteria.

### Information sources

The MEDLINE database (OVID interface, 1946 onwards) and the EMBASE database (OVID interface, 1974 onwards) were both searched to account for variability in indexing between databases [19,20]. The Cochrane database was then searched for any systematic reviews from which any relevant articles were identified [21]. The PROSPERO database of systematic review protocols was also searched for relevant protocols [22]. The electronic database search was complemented by searching for trials on the International Clinical Trials Registry Platform, clinicaltrials.gov and The Pan African Clinical Trials Registry [23–25]. Unpublished studies were identified through a grey literature search on the System for Information on Grey Literature in Europe database [26]. To conclude, a citation search of included papers was carried out to ensure literature saturation.

### Search strategy

Medical Subject Headings (MeSH) and terms relating to Ebola therapies since 2014 were used to create a search strategy. This was developed by the Principal Investigator with advice from supervisors and a librarian experienced in developing search strategies. The search strategy for MEDLINE can be found in S4 Appendix. EMBASE uses Emtree subject headings whereas MEDLINE uses MeSH headings and so the MEDLINE search strategy cannot be transferred over to Embase without alteration [27]. Therefore, a similar strategy was used for EMBASE but modified for differences in subject headings between the databases. These modifications should not have implications on the articles chosen for inclusion in this review [27]. The strategies were peer reviewed by a fellow intercalating student who advised to either broaden or reduce their comprehensiveness as necessary. After the literature search on MEDLINE and EMBASE, the other information sources listed in the previous section were examined using ‘Ebola’ as the sole search term. These other information sources contained a relatively small number of records which were all be assessed for inclusion and so there was no need to create a narrower search strategy as in MEDLINE and EMBASE search strategies.

### Study selection

#### Data management

All literature search results were loaded onto a Microsoft Excel spreadsheet. Two researchers independently assessed the results for duplicate studies by comparing author names on studies as well as abstracts of studies. If studies appeared to be duplicates, they were read in full to determine if they were identical reports one of which could then be excluded.

#### Selection process

To screen for inclusion, the title and abstract of each study was assessed independently by two researchers against eligibility criteria. Full text copies of articles were then accessed and a check for eligibility was repeated. Any disagreements were noted and were discussed with a supervisor who decided if a paper should be included. Reasons for exclusion were recorded and a record was kept of the extent of agreement at each stage of the selection process.

### Data collection process

Data extraction was carried out by a single researcher with independent verification provided by a supervisor who checked the extraction forms for accuracy. The data extraction forms can be found in S5 Appendix. Where required information appeared to be unreported, the trial authors were contacted via email for up to three attempts.

### Data items

Data was extracted from eligible studies and uploaded onto an excel spreadsheet. Data comprised specific study characteristics; most importantly, on the extent to which the study design and preliminaries reflected the ethical criteria laid out in the three research ethics frameworks. These included whether the trial was approved by a research ethics committee and ten areas which amalgamated criteria contained in the three frameworks. These criteria are summarised in Table 1.

**Table 1:** Simplified table of the ten sections that make up the three ethical frameworks. Sections are coloured **BLUE** if a similar section is present in all three frameworks, **GREEN** if they appear in two frameworks and **YELLOW** if they are only in one framework.

Criteria Number	WHO	Emanuel et al.	MSF
**1**	**Scientific design and conduct of the study**	**Scientific validity**	• **What is the research question?** • **Why is it important?** • **How is the methodology and proposed analysis appropriate given the research question(s)?**
**2**	**Risks and potential benefits**	**Favourable risk-benefit ratio**	• **What are the anticipated harms and benefits?**
**3**	**Protection of research participants’ privacy and confidentiality**	**Respect for participants**	• **How do you plan to protect confidentiality?** • **How do you plan to access, store and distribute any collected biological material?** • **What will happen when the research is either stopped or is complete?** • **How will the findings be disseminated?** • **How will the findings be implemented?**
**4**	**Informed consent process**	**Informed consent**	• **What are your plans for obtaining consent?**
**5**	**Community considerations**	**Collaborative partnership, social value**	• **What is the context in which the research will be conducted?** • **How has this influenced the research design?** • **Are there any other parties involved in the research?** • **What potential interests of these parties might conflict with MSF’s mission and values?**
**6**	**Selection of study population and recruitment of research participants**	**Fair participant selection**	** **
**7**	**Inducements, financial benefits and financial costs**	** **	** **
**8**	** **	**Independent review**	** **
**9**	** **	** **	**Are all relevant resources for the research secured?**
**10**	** **	** **	**Have the research staff the relevant training and protection?**

### Risk of bias in individual studies

As the majority of studies have not yet been published, it was decided that no formal assessment for risk of bias would be carried out. Instead, information on study conduct was extracted and presented as part of the results. The discussion will outline whether each choice of study design was appropriate given the exceptional circumstances of the Ebola outbreak.

### Summary measures

A systematic narrative analysis was carried out to analyse the extent to which studies met ethical criteria. Any answers missing data or data of concern will be described in the results and elaborated on in the discussion.

### Risk of bias across studies

We did not measure for the cumulative quality of the studies as this is not relevant to our review.

## Results

### Study selection

**Fig 1:**
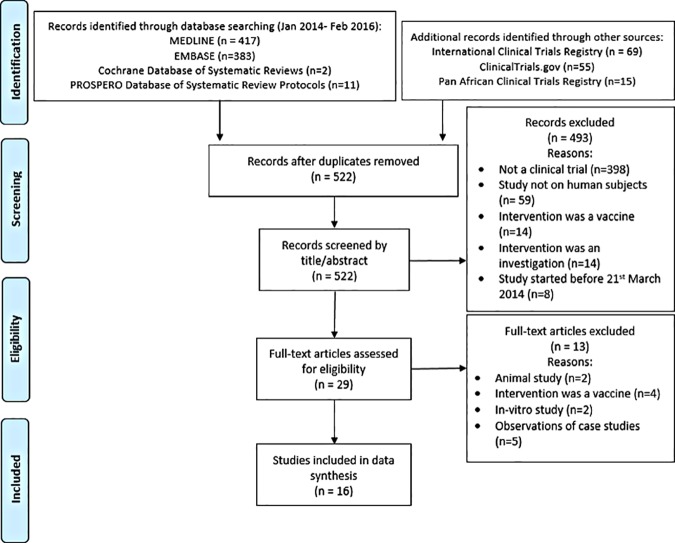
PRISMA Flow Diagram.

We identified 952 records by systematically searching MEDLINE (n = 417), EMBASE (n = 383), Cochrane Database of Systematic Reviews (n = 2), the PROSPERO Database of Systematic Review Protocols (n = 11) and three trial registries (n = 139). After removing duplicate articles, a total of 522 non-duplicate records were identified. Records were then screened by title and then abstract. Due to the specific study and report characteristics stated in the eligibility criteria, we were able to exclude 493 articles at this point. The full texts of 29 articles were then read and, of these, 13 were excluded. Reasons for exclusion at each stage are stated in the PRISMA Flow Diagram (see Fig 1).

Initially, the two researchers had an 84% degree of agreement in the selection of eligible articles. After meeting, degree of agreement increased to 88%. Following a subsequent meeting with a supervisor, the extent of agreement reached 100%. In total, 16 studies were included in the data extraction and synthesis [28–43]. 13 studies were identified through the trials registry search alone whilst the remaining three were published on MEDLINE and EMBASE databases. Contact details were available for 15 studies [28–32,34–43]. Authors were emailed up to three times with a request for missing information. Replies were received for 11 of the studies (69%) [28–30,35–39,41–43].

### Study characteristics

Tables 2, 3 and 4 list the characteristics of the population, intervention, comparator and primary outcome of each study as well as details on setting, sample size, study design, trial status and publication status. The total number intended number of participants in the studies is approximately 1800 patients although actual enrolment is likely to be less as many trials were initiated as the incidence of Ebola was decreasing. As of the 27^th^ October 2016, only four of the trials had published results [28,29,39,41]. Eight studies have been completed [28,29,32–34,36–38], one is still recruiting [40], three others are enrolling by invitation only [35,42], one has been withdrawn [30], and one is not yet recruiting patients [31].

**Table 2:** Individual study characteristics for studies 1–6.

Study Number	Title	Author(s)	Current Status	Results published?	Study design	Sample size	Population	Intervention (plus standard care)	Comparison	Primary Outcome	Setting
**1**	**Emergency Evaluation of Convalescent Plasma for Ebola Viral Disease (EVD) in Guinea [28]**	van Griensven J et al.	Completed	Yes	Emergency, phase 2/3, safety/efficacy study, open-label, non-randomized clinical trial, single-arm, historical controls used	102 (target in protocol was 102). 84 included in primary analysis	Persons with PCR- confirmed Ebola virus. No minimum age.	Convalescent Plasma	Historical controls receiving standard care alone	Mortality at day 14	Guinea
**2**	**Efficacy of Favipiravir Against Ebola (JIKI) [29]**	Malvy D et al.	Completed	Yes	Non-randomised, phase 2 efficacy study, single group assignment, open label, treatment trial	126 (target in protocol was 126)	Persons with PCR-confirmed Ebola virus, >1 year old, excluding pregnant women	Favipiravir	Historical controls receiving standard care alone	Mortality at day 14	Guinea
**3**	**Clinical Study to Assess Efficacy and Safety of Amiodarone in Treating Patients With Ebola. Virus Disease (EVD) in Sierra Leone. (EASE) [30]**	Strada G et al.	Withdrawn	No	Randomised, efficacy study, parallel assignment, open label, phase 2/3 treatment trial	0 as study withdrawn (target in protocol was 110)	Persons with confirmed Ebola virus, >2 years old, excluding pregnant women	Amiodarone	Best supportive care alone	All-cause mortality at day 10	Sierra Leone
**4**	**Multiple Treatments for Ebola Virus Disease (EVD) [31]**	Griffiss J et al.	Not yet recruiting	No	Randomised, safety/efficacy study, parallel assignment, open label, phase 1/2 treatment trial	0 as study not yet recruiting (target in protocol was 150)	Persons with confirmed Ebola virus, >6 months old.	One of: 1) atorvastatin and irbesartan, 2) azithromycin, 3) sunitinib and erlotinib, 4) IV fluids and laboratory testing	The other three interventions being compared	Death by 14 days	Sierra Leone
**5**	**Putative Investigational Therapeutics in the Treatment of Patients With Known Ebola Infection [32]**	McConnell R et al.	Completed	No	Randomised, safety/efficacy study, parallel assignment, open label, phase 1/2 treatment trial	72 (target in protocol was 333)	Persons with PCR- confirmed Ebola virus. No minimum age.	ZMapp	Optimised standard of care alone	Mortality at day 28	Guinea, Sierra Leone and Liberia
**6**	**Efficacy of Favipiravir Against Severe Ebola Virus Disease [33]**	Zhong W et al.	Completed	No	Randomised, safety/efficacy study, parallel assignment, open label, phase 2 treatment trial	77 (target in protocol was 77)	Persons with clinical diagnosis of Ebola virus and positive blood viral RNA detection, 13 to 75 years old. Pregnant or breast-feeding women excluded.	Favipiravir	WHO-recommended therapies alone	Case fatality rate at day 14	Sierra Leone

**Table 3:** Individual study characteristics for studies 7–11.

Study Number	Title	Author(s)	Current Status	Results published?	Study design	Sample size	Population	Intervention (plus standard care)	Comparison	Primary Outcome	Setting
**7**	**Clinical Trial to Evaluate the Efficacy and Safety of Convalescent Plasma for Ebola Treatment [34]**	Brown J et al.	Completed	No	Non-randomised, safety/efficacy study, open label, single group assignment, phase 1/2 pilot treatment trial	6 in total (4 in intervention arm, 2 in control arm). Target in protocol was 70.	Persons with PCR-confirmed Ebola virus, >18 years old, excluding pregnant women	Convalescent Plasma	Optimised standard of care alone	Change in viral load and Ebola Virus antibody levels	Liberia
**8**	**A Prospective, Open Label, Phase 1 Safety Study of Passive Immune Therapy During Acute Ebola Virus Disease Using Transfusion of INTERCEPT Plasma Prepared From Volunteer Donors Who Have Recovered From Ebola Virus Disease [35]**	Winkler A et al.	Enrolling by invitation	No but publication plan is in development	Non-randomised, safety/efficacy study, single group assignment, open label, phase I treatment trial	Enrolling by invitation (target in protocol was 12). Ebola survivors have consented to donate but no one enrolled to receive INTERCEPT plasma transfusion.	Persons with PCR- confirmed Ebola virus	INTERCEPT plasma	None	Proportion of subjects who survive Ebola (through hospital discharge up to one year)	United States
**9**	**Treating the Host Response to Ebola Virus Disease with Generic Statins and Angiotensin Receptor Blockers [36]**	Fedson D et al.	Completed	No (authors state that health officials in Sierra Leone have not released reports of treatment results)	Treating patients consecutively.	Approximately 100 patients were treated consecutively	Persons with confirmed Ebola virus	Atorvastatin and irbesartan	Historical controls receiving standard care alone	Mortality	Sierra Leone
**10**	**Efficacy of Favipiravir (T-705) in patients infected with Ebola virus in Sierra Leone: a preliminary clinical trial [37]**	Jiafu J et al.	Completed	No	Non-randomised, double-blind trial. No trial phase stated.	According to protocol: 85 in control group, 39 in intervention group	Persons with PCR- confirmed Ebola virus, aged >9 and <66 years old. Excluding pregnant women.	Favipiravir	WHO-recommended therapies	Death and survival	Sierra Leone
**11**	**Convalescent plasma for early Ebola virus disease in Sierra Leone [38]**	Semple C et al.	Completed	No	Emergency, non-randomized safety/efficacy study, phase 2/3, open-label clinical trial, control receives Ringer's lactate infusion.	4 patients (3 in intervention arm). Completed but results not yet published (target in protocol was 300)	Persons with PCR- confirmed Ebola virus, all ages.	Convalescent Plasma	Single intravenous bolus of Ringer's Lactate	All-cause mortality at day 14 post intervention	Sierra Leone

**Table 4:** Individual study characteristics for studies 12–16.

Study Number	Title	Author(s)	Current Status	Results published?	Study design	Sample size	Population	Intervention (plus standard care)	Comparison	Primary Outcome	Setting
**12**	**Rapid Assessment of Potential Interventions & Drugs for Ebola (RAPIDE)–TKM [39]**	Horby P et al.	Closed to recruitment: follow up continuing	Yes	Non-randomised, safety/efficacy study, single arm, open label, phase 2, treatment trial historical controls used.	Follow up continuing (target in protocol was 100)	Persons with PCR- confirmed Ebola virus, aged 0 to 99 years. Pregnant women and those aged under 18 initially excluded	TKM-100802	None	Mortality at day 14	Sierra Leone
**13**	**Treatment of Ebola virus disease with TCM: a prospective clinical study [40]**	Guo Y et al.	Recruiting	No	Observational, case series assessing efficacy of Traditional Chinese Medicine versus symptomatic treatments using western medicine. No trial phase stated.	Still recruiting (target in protocol was 30 in intervention group, 30 in control)	Persons with clinical diagnosis of Ebola virus, aged 18 to 65 years old	Traditional Chinese medicine: Qingwenbaidu decoction plus Xuebijing Injection	Standard care alone	Mortality	Sierra Leone
**14**	**Rapid Assessment of Potential Interventions & Drugs for Ebola (RAPIDE) - BCV [41]**	Horby P et al.	Closed to recruitment: follow up complete	Yes	Non-randomised, safety/efficacy study, single arm, open label, phase 2, treatment trial historical controls used.	Awaiting publication of results (target in protocol was 140)	Persons with PCR-confirmed Ebola virus, aged >2 months old. Pregnant women excluded.	Brincidofovir	Historical controls receiving standard care alone	Mortality at day 14	Liberia
**15**	**Investigation on the efficacy and safety of favipiravir in patients who are infected or strongly suspected of being infected with Ebola virus [42]**	Kato Y et al.	Enrolling by invitation	No	Non-randomised, uncontrolled, efficacy/safety study, single-arm, open-label phase 2 trial	Enrolling by invitation (target in protocol was 5)	Persons with PCR-confirmed Ebola virus or has developed symptoms in line with Ebola diagnosis and meets inclusion criteria	Favipiravir	None	Patient survival at the end of the study	Japan
**16**	**A pilot study to evaluate the safety and efficacy of interferon beta-1a for the treatment of patients with Ebola virus [43]**	Fish E et al.	Not yet recruiting	No	Non-randomised, safety/efficacy study, pilot phase 1/2, single-arm using historical controls.	Target in protocol was 30–50	Persons with suspected or confirmed Ebola, aged 18 to 69 years old	Interferon beta-1a	Historical controls receiving standard care alone	Clearance and/or reduction of viral RNA from day 1 to day 10	Guinea

#### Comparison of ethical frameworks

Table 1 shows the key areas of the ethical frameworks designed by the WHO, MSF and by Emanuel et al. respectively [14–16]. Five of the ten distinct ethical areas that this review identified appeared in all three frameworks. These can be summarised under the headings: scientific design, conduct and validity of the study, risks and potential benefits, protection of research participants’ privacy and confidentiality, process for gaining informed consent and community considerations. One area, ‘selection of study population and recruitment of research participants’, was included by the WHO and Emanuel but not MSF. The four remaining areas were included in only one of the three frameworks. The section on ‘inducements, financial benefits and costs’ was taken from the WHO framework, ‘independent review’ was specified in Emanuel’s paper whilst ‘resources for the research and protection’ and ‘training of research staff’ were contained within the MSF ethical guidelines.

### Results of individual studies

Data on each study was extracted on the following eleven criteria which indicate the extent to which studies adhered to ethical guidelines. These ethical criteria include the ten criteria which amalgamate the three research ethics frameworks mentioned previously as well as whether the trial was approved by a research ethics committee. This additional criterion is important as approval by a research ethics committee suggests that a study protocol has, in the opinion of the committee, adhered to research ethics guidelines which are based in part on the criteria stated in research ethics frameworks.

The data extraction sheets for each study can be found in the appendix (see S5 Appendix). Trials may not require all eleven elements to be considered as adhering to ethical guidelines. Indeed, the ethical frameworks from which these ethical criteria were drawn differ in terms of criteria prioritised and these differences will be described later. A detailed outline of the ethical frameworks by the WHO, MSF and Emanuel et al. can be found in S6 Appendix.

#### Ethical protocol

In total, fourteen trials were confirmed to have favourable ethical review through either viewing their ethical approval certificate or a declaration on the trials registry stating ethical approval had been granted {28–32,34,35,37–43]. In one trial, we could not confirm whether ethical approval had been granted and authors did not respond when contacted [33]. The authors of another study reasoned that giving approximately 100 consecutive patients atorvastatin and irbesartan constituted compassionate use and did not require ethical approval [36].

#### Appropriate scientific design, conduct and validity

Four studies used randomisation as part of their study design [30–33]. Twelve trials stated that randomisation would not occur [28,29,34–43]. Of the former, one study used an adaptive study design [31]. One study had no study design as such; but treated approximately one hundred patients consecutively. Upon completion of this study, physicians in Sierra Leone did not release reports of the treatment results and the study authors state that ‘it will be up to others to rigorously review and validate these findings’ [36]. One study is a Phase 1 clinical trial [35], four are listed as Phase 1/2 [31,32,34,43], five are Phase 2 clinical trials [29,33,39,41,42] and three were listed as Phase 2/3 trials [28,30,38]. Three studies did not state which phase they were in [36,37,40].

#### Favourable risk-benefit ratio

Fifteen studies specified that the intervention being tested would have a favourable balance of potential risks and benefits due to the high mortality rate for those who contract Ebola [28–39,41–43]. However, physicians have questioned the risk-benefit balance of a trial using amiodarone [13]. They argue that the adverse effects of amiodarone are not offset by the antiviral activity of the intervention as pre-clinical data on this is extremely limited. Moreover, mortality rates did not significantly decrease when the intervention was used previously to treat Ebola [13,30]. For one trial there was insufficient information available to assess the potential risks and benefits [40].

#### Protection of research participants’ privacy and confidentiality

Eight of the studies stated that identifiable information was protected and only accessible to research staff [28,29,35,38,39,41–43]. One trial stated that patients’ blood samples would be destroyed following testing but arrangements for other patient identifiable data were not included [37]. Insufficient information was available to warrant any definitive statements in seven of the studies [30–34,36,40].

#### Appropriate informed consent process

Fourteen trials stated that written informed consent was required to participate and specified procedures for those who did not have capacity to consent [29–35,38–43]. One trial did not reference informed consent in their inclusion criteria and authors did not respond to our requests for further information [37]. Fedson et al. did not gain informed consent from study participants [36]. This was justified on the grounds that physicians would be acting in the best interests of patients when administering the intervention under compassionate use and; therefore, consent was not necessary.

#### Collaborative partnership between researchers and community

In eight studies, the authors specified measures to communicate trial information to the local community [28,29,35,38,39,41–43]. In addition, two trials offered psychological and social support to those participating [39,41]. In one trial, Caucasian researchers were not used to inject the intervention to mitigate local fears that westerners were injecting people with Ebola [43]. Another trial ensured that consultations were held with patients and their families at four pre-defined points during the trial [29]. We were not provided with sufficient information to make any definitive judgments in eight of the studies [30–34,36,37,40].

#### Fair participant recruitment and selection

Sixteen studies stated inclusion criteria of participants with polymerase chain reaction (PCR) confirmed Ebola virus [28–43]. One of these studies also included those who were strongly suspected of having Ebola that had not been confirmed by PCR testing [42]. Nine studies allowed pregnant women to participate but excluded children aged two years old or younger [28,31,32,35,36,38,40,42,43]. One study excluded pregnant women but did not exclude children [41]. Five studies excluded older children: three excluded those under 18 years old [34,40,43], one patients under 13 years [33] and one excluded those under 9 years old [37].

#### Inducements, financial benefits and costs

In nine studies, intervention and all treatment, regardless of trial arm, was provided without cost to participants [28,29,35,36,38,39,41–43]. Two trials provided participants with mobile phones to enable follow up [39,41]. In two trials, reasonable travel expenses and compensation were offered [35,42]. Another trial stated expenses were only available to plasma donors but not those receiving the intervention [38]. In one study, social aid was provided to help patients return home or to assist with the family’s burial if a patient died [29]. In two other studies, no compensation was provided to participants [28,43]. In another study, a private donation enabled the medication to be delivered to Sierra Leone [36]. We were not provided with enough information on this area to warrant any definitive statements in seven studies [30–34,37,40].

#### Independent review

The involvement of both Research Ethics Review Boards and Data and Safety Monitoring Boards were considered when assessing independent review. One study did not undergo independent review as there was no written study protocol to be reviewed [36]. Three of the trials sought independent review from the WHO [39,41–43]. Two trials were reviewed by the U.S Food and Drug Administration and the Wellcome Trust respectively [35,38]. Three trials stated that an independent Data and Safety Monitoring Board was appointed to ensure continuous monitoring of their respective studies [28,29,38]. We were not provided with sufficient information to make statements in seven studies [30–34,37,40].

#### Resources for the research

Fourteen studies gave an institution as their primary sponsor; responsible for the management, financing and legal liabilities of the trial [28–35,37–42]. One trial gave an individual as their sponsor [43]. Seven trials specified an additional organisation also responsible for the funding of the trial [31,34,38,39,41–43]. One study did not have financial support from any organisations [36]. Instead, one of the authors made a private donation to bring the intervention (irbesartan and atorvastatin) into Sierra Leone [36].

#### Protection and training of research staff

Four studies were conducted in hospitals run by MSF where staff members are required to meet specified standards of training [28,29,39,41]. In two other trials, staff received specialist training to provide convalescent plasma to patients [35,38]. One trial ensured all researchers received training on infection prevention and control [42]. In another trial, twelve Guineans were trained to international standards for conducting clinical trials [43]. One study did not provide additional training for research staff [36]. We were not provided with sufficient information to make statements in seven studies [30–34,37,40].

## Discussion

### Summary of evidence

In total, eight studies appeared to fully comply with all eleven ethical criteria [28,29,35,38,39,41–43]. The other eight studies had at least one criteria where there was not enough information available to draw any conclusions [30–34,36,37,40]. In four studies there was ambiguity regarding the information provided for at least one ethical area [33,36,37,40]. In two studies there were ethical concerns regarding the information provided in relation to at least one ethical criteria [30,36]. Table 5 categorises each study’s ethical trial performance in the ten ethical areas into a traffic light system to help illustrate to what extent each trial complies with the ethical criteria.

**Table 5:** Depicts to what extent each trial complies with the ten ethical criteria contained within the three research ethics frameworks.

	Additional Criterion	The ten criteria which amalgamate the three research ethics frameworks									
Study number	Ethics approval	Study design	Risk-benefit ratio	Confidentiality	Consent	Community collaboration	Recruitment	Financial benefits	Peer Review	Funding	Research training
**1**											
**2**											
**3**											
**4**											
**5**											
**6**											
**7**											
**8**											
**9**											
**10**											
**11**											
**12**											
**13**											
**14**											
**15**											
**16**											

An additional column (ethics approval) illustrates which trials were judged by a research ethics committee to be adhering to ethical trial conduct.

Key:

**Green**—Study fully complies with criteria.

**Amber—**Ambiguity over whether criteria was met.

**Red**—Ethical concerns regarding criteria.

**Grey**—Insufficient information to draw conclusions.

The rest of this discussion will assess whether the ethical concerns stated above were appropriate given the exceptional circumstances of the Ebola outbreak.

According to the 2013 Declaration of Helsinki and similar guidelines, trials that are undertaking research on human participants are required to submit a trial protocol to research ethics committee(s) for review and approval [44,45,46]. For two studies, we were unable to find evidence of ethical review:

1. Zhong et al., did not state on their clinicaltrials.gov page that their trial had been reviewed by an ethics committee [33]. No contact details were provided and we were unable to find these independently via an internet search. We could not, therefore, contact the researchers for clarification.
2. Fedson et al. argued that ethical review was not required as they were not conducting research as patients were given atorvastatin and irbesartan under compassionate use [36].

Providing drugs to treat patients on compassionate grounds, as is the case for Fedson et al, has occurred throughout the Ebola epidemic [47,48]. The WHO argued that this was acceptable provided the clinical data was collected and shared [49]. However, the guidelines for trialling an intervention under compassionate use are still less vigorous than a clinical trial requiring ethical approval [50,51]. Therefore, it is important to determine what differentiates compassionate use from research. WHO define compassionate use as the ‘use of an intervention outside of a clinical trial’ [49]. Furthermore, the WHO reasoned that ‘compassionate use’ may be an inaccurate term in circumstances when an untested intervention is given and data on its efficacy is systematically collected from individual use. In light of this, in October 2014, the WHO Ethics Working Group suggested that the term ‘compassionate use’ should be replaced by the phase ‘monitored emergency use of unregistered and experimental interventions (MEURI)’ in an attempt to better define the often blurred boundary between clinical trials and compassionate use [49].

The WHO definition of compassionate use presents the problem of what defines a clinical trial. A widely accepted definition by the International Committee of Medical Journal Editors states that a clinical trial is ‘any research study that prospectively assigns human participants or groups of humans to one or more health-related interventions to evaluate the effects on health outcomes’ [52]. Defining research, the US Department of Health defines it as ‘a systematic investigation…designed to develop or contribute to generalisable knowledge’ [53]. In the study by Fedson et al there was a systematic process to provide over one hundred consecutive patients a novel treatment regime (irbesartan and atorvastatin) and systematically collect data on subsequent mortality rates [36]. Given this information and the aforementioned definitions, it could be argued that the limits for what constitutes ‘compassionate use’, and therefore does not require prior ethical approval, have been strained.

The use of randomisation in clinical trials has been contentious in the current Ebola outbreak [54–59]. Both randomised and non-randomised study designs were used by the reviewed studies. The WHO guidance suggests that all study designs, whether randomised or not, should be considered provided standards for human research ethics are adhered to [49]. An argument against randomisation is that it may not be acceptable to the local community who, against the background of Ebola’s high mortality rate, may not understand why some people are being given a potentially promising treatment whilst others are not [54,55]. On the other hand, randomising minimises allocation bias and reduces confounding factors [56,57,58]. Arguably, in the context of this Ebola outbreak, any reasonable and robust study design, sensitive to local conditions and meeting other ethical standards might be regarded as acceptable [49,59]. Given that results published so far are from non-randomised trials, there is insufficient evidence to draw any firm conclusions about the relative merits of each study design.

The majority of studies demonstrated a clearly favourable balance of potential risks and benefits given the high fatality rate of Ebola. Concerns have been expressed about the balances of risks and benefits in the proposed trial by Strada et al. using amiodarone [30]. It was contended that amiodarone is a potentially toxic drug with side-effects including thyroid toxicity and abnormal ECG changes in hypokalaemic patients [13]. Such risks, it was argued, would not be outweighed by the benefits of amiodarone as pre-clinical data on the antiviral activity of amiodarone is very limited. Moreover, of the 65 patients who were previously given amiodarone under compassionate use, 63% died compared to around 50% who were receiving best supportive therapy at the Ebola treatment unit where the trial took place [13]. This trial did secure ethical approval from the Sierra Leone research ethics committee who presumably formed a contrary view about the potential balance of risks and benefits [30]. The importance of local review, even at times of crisis, cannot be overstated: more support may be needed to bolster local research ethics review services in disaster settings to provide robust, timely scientific and ethical review.

There were inconsistencies across trials regarding their exclusion criteria for both pregnant women and children. On the one hand, children should not be excluded from a trial purely on the basis that they may have diminished autonomy as the informed consent process can be adapted to accommodate this and appropriate measures taken to ensure confidentiality is respected [60]. On the other hand, studies may have excluded children on the basis that the intervention may have adverse outcomes on a child’s growth and development [61]. The usual norm of requiring that interventions are trialled on adults before children may not protect the interests of children in such extreme circumstances where the entire affected population might be regarded as vulnerable [46]. Similarly, regarding the exclusion of pregnant women, some studies may have included pregnant women on the basis that the potential benefit to the mother may outweigh potential teratogenic effects of the intervention on her unborn foetus [62]. Moreover, the death of a pregnant woman inevitable affects, if no seals, the survival prospects of the foetus. Given that trials registries do not collect information on the reasons behind exclusion criteria, conclusions about some researchers excluded pregnant women or children are difficult to make.

Five studies’ originators did not reply to requests for further information. In these cases, data on the ethical criteria was limited to information in the public domain. This was predominantly on trials registries where there is no requirement to report information relating to the following five ethical criteria:

1. Protection of research participants’ privacy and confidentiality
2. Collaborative partnership between researchers and community
3. Inducements, financial benefits and costs
4. Independent review
5. Protection and training of research staff

This is surprising given the importance of these five criteria in promoting ethical conduct. For instance, Emanuel et al. state that independent review of research protocols enables greater accountability and reduces concerns surrounding authors’ potential conflicts of interests [16]. Similarly, WHO guidance states that communicating and engaging with local communities is of paramount ethical importance [14]. Going forward, it would be useful if there were obligatory sections in all trial registries on how these ethical criteria would be met and these sections should be added to the WHO Trial Registration Data Set [63].

#### Comparison of ethical frameworks

The 2014–2015 Ebola outbreak was unprecedented in its magnitude and placed huge strains on fragile healthcare systems in the affected communities [64]. This, coupled with the absence of any specific treatment for Ebola owing to the relative lack of research carried out in previous outbreaks, contributed to the exceptional circumstances of this disaster [65]. In this respect, it is important to acknowledge that some ethical concerns may need to be prioritised over others for clinical trials to take place in such circumstances.

The three research ethics frameworks are well established yet each specifies at least one ethical area that is missing from the other two frameworks as depicted in Table 1. There are five criteria which are not present in all three frameworks. The authors of these frameworks present logical arguments for why these criteria are essential to fully assess the ethical conduct of a trial. Therefore, as a minimum standard for research ethical conduct in emergency disaster settings, this review suggests that all trials should conform to the ten ethical areas as summarised below.

These ten criteria are:

1. Appropriate scientific design, conduct and validity
2. Favourable risk-benefit ratio
3. Protection of research participants’ privacy and confidentiality
4. Appropriate informed consent process
5. Collaborative partnership between researchers and community
6. Fair participant recruitment and selection
7. Inducements, financial benefits and costs
8. Independent review
9. Resources for the research
10. Protection and training of research staff

### Limitations

Authors were contacted if data was unreported and given the opportunity to provide this information. Five authors did not reply to three attempts to contact them. Information regarding the ethical conduct of these trials is, therefore, limited to material in the public domain. We did not have the resources to assess whether data from pre-clinical, phase one or phase two studies was available for studies in later phases. Similarly, the amount of data provided by the authors in correspondence varied greatly and this meant it was not always possible to assess the extent to which ethical guidelines were met. Twelve studies have not yet published results and so assessment of ethical conduct was carried out based on information on trial registries, contact with study authors or, if available, protocols submitted to ethics committees. Therefore, no conclusions can be made on the extent to which research protocols were adhered to.

## Conclusions

The majority of trials adhered to ethical guidelines during this particularly challenging epidemic. Although some deviation from standard research norms in disaster situations is widely accepted, international regulations are in place and should not be ignored [44,46]. The recommendations from this review are threefold:

Firstly, new guidelines should be generated to better define the boundaries between using medicines for compassionate use and conducting a clinical trial. This should be coupled with increased standardisation of data sets across clinical trial registries. These changes will promote greater transparency and accountability when conducting research.

Secondly, there should be greater support for local research ethics committees to provide robust, timely, scientific and ethical review in those countries affected by disasters such as Ebola.

Lastly, we propose a framework for the minimum standards for research ethics in disaster settings. The framework contains ten criteria, five of which integrate the common criteria contained within the three frameworks assessed in this review. As this was a secondary objective of the systematic review, a future review of all ethical guidance may highlight other areas of commonly used criteria as well as differences that can be explored to ensure a consensus on what a comprehensive, universally applicable guidance should contain.

## Supporting Information

S1 Appendix PRISMA checklist for systematic reviews. (DOCX)

S2 Appendix PRISMA for Abstracts Checklist. (DOCX)

S3 Appendix Systematic Review Protocol. (DOCX)

S4 Appendix Search strategy used for MEDLINE systematic search. (DOCX)

S5 Appendix Data extraction forms for the sixteen included studies. (DOCX)

S6 Appendix Detailed outline of the three ethical frameworks by the WHO, MSF and Emanuel et al. (DOCX)
