# Supplementary material for: A Systematic Review of Ebola Treatment Trials to Assess the Extent to Which They Adhere to Ethical Guidelines
Source: PLoS One. 2017 Jan 17;12(1):e0168975. doi: 10.1371/journal.pone.0168975 (PMC5240928; doi:10.1371/journal.pone.0168975)
Supplement: S2 Appendix — (DOCX) [file pone.0168975.s002.docx]

| **TITLE** | **CHECKLIST ITEM** | REPORTED ON PAGE # |
| --- | --- | --- |
| 1. Title: | Identify the report as a systematic review, meta-analysis, or both. | TITLE PAGE |
| **BACKGROUND** |  |  |
| 2. Objectives: | The research question including components such as participants, interventions, comparators, and outcomes. | 1 |
| **METHODS** |  |  |
| 3. Eligibility criteria: | Study and report characteristics used as criteria for inclusion. | 1 |
| 4. Information sources: | Key databases searched and search dates. | 1 |
| 5. Risk of bias: | Methods of assessing risk of bias. | - |
| **RESULTS** |  |  |
| 6. Included studies: | Number and type of included studies and participants and relevant characteristics of studies. | 1 |
| 7. Synthesis of results: | Results for main outcomes (benefits and harms), preferably indicating the number of studies and participants for each. If meta-analysis was done, include summary measures and confidence intervals. | 1 |
| 8. Description of the effect: | Direction of the effect (i.e. which group is favoured) and size of the effect in terms meaningful to clinicians and patients. | 1 |
| **DISCUSSION** |  |  |
| 9. Strengths and Limitations of evidence: | Brief summary of strengths and limitations of evidence (e.g. inconsistency, imprecision, indirectness, or risk of bias, other supporting or conflicting evidence) | - |
| 10. Interpretation: | General interpretation of the results and important implications | 2 |
| **OTHER** |  |  |
| 11. Funding: | Primary source of funding for the review. | 2 |
| 12. Registration: | Registration number and registry name. | 2 |

**S2 Appendix. The PRISMA for Abstracts Checklist**
