# Supplementary material for: A Systematic Review of Ebola Treatment Trials to Assess the Extent to Which They Adhere to Ethical Guidelines
Source: PLoS One. 2017 Jan 17;12(1):e0168975. doi: 10.1371/journal.pone.0168975 (PMC5240928; doi:10.1371/journal.pone.0168975)
Supplement: S3 Appendix — (DOCX) [file pone.0168975.s003.docx]

**S3 Appendix. Systematic review protocol**

**RESEARCH PROJECT PROTOCOL**

The PRISMA-P 2015 checklist will be used as a framework for this systematic review protocol as this is recommended by the BMJ (1).

**ADMINISTRATIVE INFORMATION**

1. **Title**

A systematic review of Ebola treatment trials to assess whether they adhere to ethical guidelines: protocol for a systematic review.

**Keywords**: Ebola, Treatment, Systematic Review, Ethics, Ethical Trial Performance

2. **Registration**

Our systematic review protocol will be registered with the International Prospective Register of Systematic Reviews (PROSPERO).

3. **Authors:**

a) **Contact information**

Thomas Richardson (TR), (AB), these are not the student’s real initials as this assignment must be marked anonymously. Medical Student Public Health Intercalation Degree, Medical School, College of Medical and Dental Sciences, University of Birmingham, Birmingham, UK; thr249@bham.ac.uk

Lt Col Andrew McDonald Johnston (AJ), Consultant Physician, Academic Department of Military Anaesthesia and Critical Care and Department of Military Medicine, Royal Centre for Defence Medicine, Queen Elizabeth Hospital Birmingham, Birmingham B15 2WB, UK; [andy.johnston@uhb.nhs.uk](mailto:andy.johnston@uhb.nhs.uk)

Professor Heather Draper (HD), Professor of Biomedical Ethics, Medicine, Ethics, Society and History (MESH), School of Health and Population Sciences, University of Birmingham, Birmingham, B15 2TT, UK; [h.draper@bham.ac.uk](mailto:h.draper@bham.ac.uk)

Kishaan Jeyanantham (CD), Medical Student Public Health Intercalation Degree, Medical School, College of Medical and Dental Sciences, University of Birmingham, Birmingham, UK; [KXJ217@student.bham.ac.uk](mailto:KXJ217@student.bham.ac.uk)

b) **Author Contributions**

AB is the guarantor. AJ provided the expertise regarding the 2014/15 Ebola outbreak. HD provided the expertise on the ethical issues that occur in trials that take place in epidemics. AB and AJ contributed to developing the research question. AB and KJ developed the search strategy and selected studies to be included in the review with feedback from AJ. AB developed the data extraction criteria. AB was principally responsible for producing the protocol as it is a compulsory component of their University degree but AJ and HD provided feedback on draft protocols.

4. **Amendments**

If amendments have to be made to the protocol, these will be documented with the date of the amendment and a description of the change as well as the rationale behind it.

5. **Support**

a) **Sources**

AB is able to claim back up to £500 from the University of Birmingham to support this research.

b) **Sponsor**

None

c) **Role of sponsor/funder**

This systematic review is funded by the University of Birmingham as part of AB’s intercalation degree. Up to £500 can be claimed back by AB to support the data collection, management and analysis of the study. Any other funds above £500 must be met by AB. The University will have no involvement on how the results of the study are interpreted.

**INTRODUCTION**

6. **Rationale**

The 2014 Ebola outbreak is the most widespread and fatal epidemic of Ebola in history with over 11000 deaths due to the viral haemorrhagic disease (1). This has resulted in a worldwide emergency response in order to contain the virus including a rapid increase in research and development into potential Ebola treatments and vaccines (2). However, as of November 2015, there is no licensed drug therapy against Ebola and currently recovery is dependent on doctors providing best supportive care and the patients’ immune response (3). A number of potential therapeutics have been used in compassionate care; however, the evidence for using these is often not based on any solid scientific evidence base and their safety and side effects are unknown (4). It is argued that we can deviate from normal research expectations if these deviations are justified by the circumstances of the disaster. In the case of Ebola, due to the urgent requirement to provide patients with treatment and to assess the effectiveness of interventions, the WHO in August 2014 concluded that it would be acceptable to use unregistered treatments that had shown promising results in animal models but had not been tested for safety or efficacy in humans (5). This was providing that certain criteria were met including a number of ethical requirements (6). These requirements include, but are not limited to: transparency about care, fairness, informed consent, freedom of choice, confidentiality, respect for the person, preserving dignity, risk-benefit assessment and community involvement (7).

Throughout the outbreak, a number of trials have been proposed to assess the safety and efficacy of a variety of drugs to treat Ebola. Concerns have been raised about some of these proposed trials. Firstly, whether they have been carried out according to worldwide ethical standards and, been secondly, if they have been approved by a research ethics review committee. Whilst the ethics committee cannot ensure that a trial is carried out ethically, it acts as a filter for ensuring poor research does not take place. This systematic review is most interested in the first point as independent validation- such as being approved by an ethics committee- is only one part of ensuring a study is carried out ethically. Furthermore, one could argue that without seeking approval from an ethics committee, a study could still be undertaken ethically if all the other ethical standards are upheld.

Two trials have raised particular ethical issues. Firstly, 100 consecutive patients were prescribed atorvastatin and irbesartan under compassionate use in order to treat Ebola (8). The study claimed that these patients had a 2% mortality rate- normally untreated Ebola has a mortality rate of around 50% (9). Whilst these results seem promising, this mortality rate was non-verified. Furthermore, there was no formal documentation of the patients’ treatment results and many of the papers conclusions are based on unpublished observations. Lastly, the methods suggest that signed informed consent was not obtained to give patients these drugs and the actual consent process is not stated.

The second study which raises ethical questions is registered on the Pan African Clinical Trials Registry but has not taken place nor been approved by any Ethics Review Committee (10). The study proposes assessing the efficacy and safety of amiodarone in Ebola patients (11). At least 132 patients will be randomised to receive either amiodarone and best supportive care or best supportive care alone (12). Previously, amiodarone had been given to 65 patients with Ebola as a compassionate therapy of which 63% died. The authors acknowledge that there is no evidence to suggest that amiodarone had any effect on these patients (13). Furthermore, clinicians have argued that amiodarone’s potentially toxic side-effects mean it should not be given off-licence to treat Ebola without the patient’s consent (14). Given that no method of taking consent is stated in the protocol and that amiodarone is not on the WHO list of essential medicines to combat Ebola, the evidence base for using amiodarone in a trial of this sort appears speculative at best (15).

According to the most recent WHO report published in October 2015 on research and development of Ebola therapies, there are fifteen Ebola treatment trials currently in progress (16). Two of these raise serious ethical concerns. A systematic review of all trials aimed at determining the extent to which they have complied with international and local ethical standards for research is therefore timely.

If trials were found to have not followed these ethical principles, then this will illustrate a greater need for transparency and accountability from researchers, not just in the current Ebola outbreak but in any situation where a novel infectious disease precipitates a medical emergency. As most countries have been declared Ebola free (as of the 5^th^ December 2015), studies are not currently able to recruit patients with Ebola and therefore the results from this systematic review could allow trials to be amended before the next disease outbreak.

7. **Objectives**

The aim of this systematic review is to examine all Ebola treatment trials that have been conducted during the 2014 outbreak and systematically review whether they satisfy international ethical criteria for trial conduct. These criteria will be taken from three published ethical frameworks; the WHO (17), an NGO (MSF) (17) and a framework by Emanuel, Wendler and Grady which has been designed specifically for use in developing countries (17). The proposed review will have the following objectives:

**Primary Objective**

To determine to what extent each trial meet criteria specified in the three research frameworks for ethical trial conduct.

**Secondary Objective**

To compare the three ethical frameworks and to suggest how they be modified and improved.

**METHODS**

8. **Eligibility criteria**

**Study characteristics:**

*Study design*: The review will include all study designs. This is because the purpose of the systematic review is not to assess the choice of design but rather adherence to ethical conduct. Studies can be in any phase and will eligible for inclusion even if it is registered but participant recruitment does not take place. This is because there are still ethical implications for starting a trial and not completing it. There will be no restriction on sample size.

*Participants*: We will include studies that have taken place since Ebola was first identified in on the 21^st^ March 2014. We will include studies on participants, both adults and children, who are suspected of having Ebola regardless of what country they are from.

*Interventions*: All studies must be using interventions to treat patients with Ebola. Because the nature of the systematic review is to assess for whether studies followed ethical criteria as opposed to the efficacy of Ebola therapies, there will be no limitations on which interventions will be included. Studies will not be excluded if there is no control group and only collected outcome data for the intervention. The WHO currently lists 15 treatment regimens which are being trialled to treat for Ebola. The interventions include: ZMapp, Favipiravir, TKM-130803, Brincidofovir, Convalescent Plasma, Convalescent Blood, Interferon beta 1a, BCX4430, MIL77, Amiodarone, Artesunate-amodiaquine, Atorvastatin + irbesartan (+/- clomifene), FX06, ZMAb and Lamivudine (18).

*Comparators*: All comparators will be included as some control arms should be subject to the same standards as intervention arms. Controls in many studies include historical controls as all participants in the trial have received the treatment. This is a similar case for treatment given for compassionate use where there is no control group. Other controls include the standard clinical care in the country the trial is set in.

*Outcomes*: We will include trials regardless of outcomes reported as the outcomes themselves need to be assessed to see whether they are ethically acceptable. The majority of phase two trials use viral load as their primary outcome whilst treatments used for compassionate use often have mortality as a primary outcome. We will consider trials whether they are assessing the efficacy, safety, tolerability or pharmacokinetics of an intervention.

*Timing*: Studies will be selected on the basis that they have been started since the 2014 Ebola outbreak began. There will be no restrictions on the length of follow up of studies.

*Setting*: There will be no restrictions on the setting.

**Report characteristics:**

*Geographical location*: We will include studies from any country as this will enable us to analyse whether there is a difference between the ethical performances of studies carried out in developing countries versus developed countries.

*Language*: We will report all articles written in English but we do not have the resources to translate the full text of non-English studies. Therefore, all non-English studies will be listed in a separate section within the appendix but will be ineligible for inclusion in the data analysis.

*Publication status and date*: We will include all research published from 2014 onwards. We will also include any unpublished data whether that be in full or abstract form as unpublished data may be less likely to have satisfied ethical criteria.

9. **Information sources**

This systematic review search will use Medical Subject Headings (MeSH) and terms relating to Ebola therapies since 2014 to develop a search strategy. The literature search will be limited to studies on human subjects. We plan to search the MEDLINE database (OVID interface, 1946 onwards) (19) as well as the EMBASE database (OVID interface, 1974 onwards) (20) to account for variability in indexing between databases. We will also search the Cochrane database for any relevant reviews and identify any relevant papers from them (21). The PROSPERO database of systematic review protocols will also be searched for relevant systematic review protocols (22). The electronic database search will be complemented by searching for trials on the International Clinical Trials Registry Platform (23), clinicaltrials.gov (24) and The Pan African Clinical Trials Registry (25). The October 2015 public report by the WHO on Ebola Research and Development: Landscape of Clinical Candidates and Trials listed 15 trials of Ebola therapies, all of which will be assessed for inclusion in the systematic review (26). Unpublished studies will be identified through a grey literature search on the System for Information on Grey Literature in Europe database (27). Following the literature search, a citation search of included papers will be carried out in order to ensure literature saturation.

10. **Search strategy**

Medline and EMBASE databases will be searched using a specific search strategy. This strategy will be developed by AB with advice from HD and AJ as well as a librarian experienced in developing search strategies for systematic reviews. This librarian will not be associated with the project in any other capacity. A draft search strategy for MEDLINE has been included in the appendix (see Appendix 1). This will be similar for EMBASE but modified for any differences in subject headings between the two databases.

The search strategy will include studies written in non-English language; however, only English studies will be reviewed due to resource constraints on translating foreign language papers. There will be no restriction on study design but all studies must be related to the 2014 Ebola Outbreak and so studies must have been published after the 21^st^ March 2014 which is the date the first case of Ebola was formally identified.

Following the literature search on Medline and EMBASE, the Cochrane Library and PROSPERO databases will be searched for relevant systematic reviews or review protocols. The clinical trial registries listed in section 9 will then be searched as well as a grey literature database. Following this, a citation search of all papers identified will be carried out. The literature search strategy will be peer reviewed by KJ who will advise to either broaden or reduce the comprehensiveness of the strategy as necessary.

11. **Study records**

a) **Data management**

All literature search results will be recorded and loaded onto a Microsoft Excel spreadsheet. At this stage, AB and KJ will independently assess for duplicate studies by comparing author names on studies as well as abstracts of studies. If studies appear to be duplicates, they will be read in full to see if they are duplicate reports of the same study which will introduce bias or multiple reports of the same study (28). Duplicate reports of the same study will then be excluded.

b) **Selection process**

To screen studies for inclusion, the title and abstract of each study will be assessed independently by AB and KJ to ensure they meet criteria outlined in section 3. Full text copies of these papers will then be obtained and a check for eligibility criteria will be repeated. AB and KJ will then meet and any discrepancies between paper inclusions will be noted and discussed. If questions about eligibility cannot be resolved, we will present our reasoning to AJ who will decide whether a paper should be included. Reasoning for excluding trials will be recorded and a record will be kept of the extent to which AB and KJ agree at each stage of the selection process. Studies will not be excluded on the basis of methodological quality as this has no effect on the outcomes of this systematic review. When assessing for study inclusion, neither AB, KJ nor AJ will be blind to the paper authors, their institutions or the journal they were published in.

c) **Data collection process**

Single data extraction will be carried out by AB with verification provided by AJ. The data extraction form has been attached as part of the appendix (see Appendix 2). If any of the information we require appears to be unreported, we will initially contact the authors via email. We will attempt this up to three times after which we will contact the national research committee of the country in which the study was registered for a copy of the research proposal of the study which should contain the missing information, relating to the ethics of the study.

12. **Data items**

Data extracted will include but is not limited to the study title, author, study design, country/location of trial, study population and demographics, sample size, start and end date, publication status, trial status, intervention and control details, and all reported outcomes that relate to Ebola treatment. In order to answer our question about whether research followed standard ethical principles, data regarding the ethics of the study will also be extracted. This will include whether the trial was approved by a research ethics committee and all data related to answering principles set out in each of the three ethical frameworks (29).

13. **Outcomes and prioritisation**

The primary outcome of this review is to assess to what extent each trial complies with the three research ethics frameworks which set out ethical criteria to follow when carrying out research. Any areas which do not comply with the frameworks or that are missing entirely will be addressed in the discussion. If, for example, patients did not give fully informed written consent, we will assess what impact this has on the ethical performance of the study. It may be that written consent could not be obtained due to the patient being unconscious. This would be less unethical than if written consent was not obtained as the patient refused to consent to the treatment.

Our secondary outcome is that we will compare the three ethical frameworks to see whether there are differences between the questions they cover and will discuss why these differences exist. This will enable us to make recommendations on how to improve future frameworks to maximise the ethical integrity of research.

14. **Risk of bias in individual studies**

Each study will be assessed for risk of bias as scientific validity forms part of the criteria for ethical conduct. The validated tool used to assess this will depend on whether the trial is randomised or not. Randomised trials will be assessed for risk of bias using the Cochrane Collaboration Tool for Assessing Risk of Bias (30) whilst cohort and case-control studies will be assessed using the Newcastle-Ottawa Scale (31). Other study designs will not be assessed using a standardised scale as no validated scale currently exists. In this case, common biases in each study design will still be assessed but not using a standardised method.

15. **Data synthesis**

A systematic narrative analysis will be carried out in order to analyse the extent to which studies met ethical criteria. The narrative synthesis will follow the guidance as set out by Popay et al. for conducting narrative analysis in systematic reviews (32). Tables will be used to show how each study fulfilled each of the criteria of the three ethical frameworks. If there are any answers missing or answers appear unethical, these will be discussed in the text. We will report on all studies regardless of trial design as the design may affect the ethical performance of a study and so need to be assessed. For example, we will analyse both randomised and non-randomised studies. Non-randomised studies are often carried out in emergencies if resources are limited where as a randomised trial may result in a higher quality study but ethically it is questionable to offer only some Ebola patients a treatment which may help them.

16. **Meta-bias**

We will not attempt to confirm whether publication bias or outcome reporting bias was present in these studies as this will not impinge on the quality of our systematic review.

17. **Confidence in cumulative evidence**

We do not plan to measure the quality of the studies we have included as ethical trial performance is not effected by the quality of the study for example by risk of bias, consistency in results or precision of results.
