# Supplementary material for: A Systematic Review of Ebola Treatment Trials to Assess the Extent to Which They Adhere to Ethical Guidelines
Source: PLoS One. 2017 Jan 17;12(1):e0168975. doi: 10.1371/journal.pone.0168975 (PMC5240928; doi:10.1371/journal.pone.0168975)
Supplement: S4 Appendix — (DOCX) [file pone.0168975.s004.docx]

**S4 Appendix. Search strategy used for MEDLINE systematic search**

| 1. Hemorrhagic Fever, Ebola/ |  |
| --- | --- |

| 2. Hemorrhagic Fever, Ebola/ |  |
| --- | --- |

| 3. limit 2 to yr="2014-Current" |  |
| --- | --- |

| 4. Therapeutics/ |  |
| --- | --- |

| 5. 2 and 3 and 4 |  |
| --- | --- |

| 6. 2 and 4 |  |
| --- | --- |

| 7. Ebolavirus/ or Hemorrhagic Fever, Ebola/ |  |
| --- | --- |

| 8. Ebolavirus/ or Hemorrhagic Fever, Ebola/ |  |
| --- | --- |

| 9. limit 8 to yr="2014-Current" |  |
| --- | --- |

| 10. Hemorrhagic Fever, Ebola/th [Therapy] |  |
| --- | --- |

| 11. Hemorrhagic Fever, Ebola/th [Therapy] |  |
| --- | --- |

| 12. limit 11 to yr="2014-Current" |  |
| --- | --- |

| 13. Hemorrhagic Fever, Ebola/dt [Drug Therapy] |  |
| --- | --- |

| 14. limit 13 to yr="2014-Current" |  |
| --- | --- |

| 15. Hemorrhagic Fever, Ebola/dt, th [Drug Therapy, Therapy] |  |
| --- | --- |

| 16. limit 15 to yr="2014-Current" |  |
| --- | --- |

| 17. Hemorrhagic Fever, Ebola/dt, th [Drug Therapy, Therapy] |  |
| --- | --- |

| 18. limit 17 to yr="2014 -Current" |
| --- |
