# Supplementary material for: A Systematic Review of Ebola Treatment Trials to Assess the Extent to Which They Adhere to Ethical Guidelines
Source: PLoS One. 2017 Jan 17;12(1):e0168975. doi: 10.1371/journal.pone.0168975 (PMC5240928; doi:10.1371/journal.pone.0168975)
Supplement: S5 Appendix — (DOCX) [file pone.0168975.s005.docx]

| **Study title** | [**Emergency Evaluation of Convalescent Plasma for Ebola Viral Disease (EVD) in Guinea**](https://clinicaltrials.gov/ct2/show/NCT02342171?term=ebola&rank=3) |
| --- | --- |
| **Study Number** | [1](https://clinicaltrials.gov/ct2/show/NCT02342171?term=ebola&rank=3) |
| **Study author(s)** | van Griensven J et al. |
| **Ethical approval** | Yes |
| **Scientific design, conduct and validity of the study** | Emergency, phase 2/3, open-label, non-randomized clinical trial, historical controls used |
| **Favourable risk-benefit ratio** | Yes, for both patients and plasma donors. Benefits to patient: Plasma transfusion may results in increased survival. If confirmed, this intervention could be scaled up and be beneficial for all Ebola patients and increase trust in medical profession from local community. Risks: Transmission of infection and adverse reactions relating to transfusion. Transfusion may not be acceptable for the local community. |
| **Protection of research participants' privacy and confidentiality** | Donors' and patients' confidentiality protected in line with international guidelines. Patient’s data coded. Any identifiable information will only be accessible by medical staff. Samples sent out of the country for testing will be coded. Other patients may see who is being transfused due to scarce resources at the Ebola treatment centres. |
| **Informed consent process** | No subject can be recruited until written informed consent has been obtained. Separate consent process for donors and patients. Two-step consent for patients. Appropriate procedures in place for minors and those with no capacity to consent including those whose lack of capacity is not permanent. Participants can withdraw at any time. Patients who withdraw will receive standard care. |
| **Community considerations and collaborative partnership** | Local communities and stakeholders were engaged with prior to the trial starting in order to ensure community acceptance. Social norms and beliefs were addressed to ensure the trial was implemented in a culturally acceptable way. |
| **Selection and recruitment of research participants** | An appropriate sample size calculation was carried out. Inclusion and exclusion criteria were appropriate and did not cause a particular group of persons to bear more than their fair share of risks in participating in the trial. Vulnerable patient groups were able to take part with safeguards in place to protect them. |
| **Inducements, financial benefits and financial costs** | Patients and donors will not receive payment for participation. Any treatments required for either intervention or standard supportive care will be provided free of charge to the patient. |
| **Independent Review** | A Data and Safety Monitoring Board was put in place before recruitment began to ensure independent continuous monitoring of the safety of the trial. Due to logistical challenges and biosafety risk, an external monitoring scheme was deemed not appropriate so instead a member of the team was given the role of 'internal monitor' |
| **Resources for the research** | The sponsor of the trial took a 'no-fault' insurance policy to cover any harms to subjects as a direct result of participating in the trials. The study could be terminated early by the Data Safety Monitoring Board. |
| **Research staff training and protection** | The study will take place at the Ebola treatment centre managed by MSF in Conakry, Guinea. All staff are trained to MSF minimum standards and will have training in transfusing convalescent plasma. |

**S5 Appendix. Data extraction forms for the sixteen included studies**

| **Study title** | [**Efficacy of Favipiravir Against Ebola (JIKI)**](https://clinicaltrials.gov/ct2/show/NCT02329054?term=ebola&rank=11) |
| --- | --- |
| **Unique study number** | 2 |
| **Study author(s)** | Malvy D et al. |
| **Ethical approval** | [Yes](https://clinicaltrials.gov/ct2/show/NCT02329054?term=ebola&rank=11) |
| **Scientific design, conduct and validity of the study** | Non-comparative, proof-of-concept, phase II trial |
| **Favourable risk-benefit ratio** | No previous study using Favipiravir to treat Ebola. Drug may increase chance of survival. In a previous trial versus influenza there were not major side effects but this trial is using higher doses. |
| **Protection of research participants' privacy and confidentiality** | All data will remain confidential and accessed and analysed only by the medical team associated with the study. Data will be identified by a unique identifier ensuring that participant data is anonymised. |
| **Informed consent process** | Informed consent is a criteria for enrolment in the study. It is given by signing consent form. If this is not possible, fingerprint or any other form of non-written consent will be documented. Witnesses may participate in consent process if given patients permission. |
| **Community considerations and collaborative partnership** | Community leaders will be targeted to inform them about the trial so they can answer questions regarding the trial from their community. To avoid misunderstanding and mistrust, patient families will be spoken to at four key time points via phone calls or face to face. |
| **Selection and recruitment of research participants** | All participants will be recruited from hospitals in Guinea. An appropriate sample size calculation was carried out. Inclusion and exclusion criteria were appropriate and did not cause a particular group of persons to bear more than their fair share of risks in participating in the trial. Vulnerable patient groups were able to take part with safeguards in place to protect them. |
| **Inducements, financial benefits and financial costs** | Social aid is included in the study budget to prepare patients to return home if declared cured or help families following a patient's death. Patients will not be paid for participation |
| **Independent Review** | Independent Data Safety Monitoring Board of seven external experts will oversee the trial. They can enforce the premature discontinuation of part or the entirety of the trial. A steering committee and scientific advisory board meet to ensure the trials remains scientifically relevant |
| **Resources for the research** | Sponsor (INSERM) has taken out civil liability insurance. Measures are put in place to support patients should the trial be terminated at any point. |
| **Research staff training and protection** | The study will take place initially at the MSF Ebola Virus Disease care centre in Gueckedou and may then expand depending on inclusion rate. All research and care personnel will be trained to international standard procedures for preventing and treating Ebola. All will be fully insured medically by the sponsor |

| **Study title** | [**Clinical Study to Assess Efficacy and Safety of Amiodarone in Treating Patients With Ebola. Virus Disease (EVD) in Sierra Leone. EASE (EMERGENCY Amiodarone Study Against Ebola)**](https://clinicaltrials.gov/ct2/show/NCT02307591?term=ebola&rank=15) |
| --- | --- |
| **Unique study number** | [3](https://clinicaltrials.gov/ct2/show/NCT02307591?term=ebola&rank=15) |
| **Study author(s)** | Strada G et al. |
| **Ethical approval** | Yes |
| **Scientific design, conduct and validity of the study** | Randomised, efficacy study, parallel assignment, open label, phase II/III treatment trial comparing amiodarone and best standard care to best standard care alone |
| **Favourable risk-benefit ratio** | Benefits: Potential increase in chance of survival. Risks: Adverse effects of amiodarone. This drug has not been tested for efficacy versus Ebola virus before and when used in compassionate use mortality did not drop significantly. |
| **Protection of research participants' privacy and confidentiality** |  |
| **Informed consent process** | Patients must consent to the trial before they are allowed to participate. |
| **Community considerations and collaborative partnership** |  |
| **Selection and recruitment of research participants** | All participants recruited from a hospital in Sierra Leone. Inclusion and exclusion criteria were appropriate and did not cause a particular group of persons to bear more than their fair share of risks in participating in the trial. Vulnerable patient groups were able to take part with safeguards in place to protect them. |
| **Inducements, financial benefits and financial costs** |  |
| **Independent Review** |  |
| **Resources for the research** | Funding is by EMERGENCY (NGO). |
| **Research staff training and protection** |  |

| **Study title** | [**Multiple Treatments for Ebola Virus Disease (EVD)**](https://clinicaltrials.gov/ct2/show/NCT02380625?term=ebola&rank=17) |
| --- | --- |
| **Unique study number** | [4](https://clinicaltrials.gov/ct2/show/NCT02380625?term=ebola&rank=17) |
| **Study author(s)** | Griffiss J et al. |
| **Ethical approval** | Yes |
| **Scientific design, conduct and validity of the study** | Adaptive, randomised, safety/efficacy study, parallel assignment, open label, phase 1/2 treatment trial |
| **Favourable risk-benefit ratio** | No previous study using Favipiravir to treat Ebola. Drug may increase chance of survival. In a previous trial versus influenza there were no major side effects but this trial is using higher doses. |
| **Protection of research participants' privacy and confidentiality** |  |
| **Informed consent process** | Inclusion criteria specifies that participant or family member/guardian must be able and willing to provide signed informed consent |
| **Community considerations and collaborative partnership** |  |
| **Selection and recruitment of research participants** | Inclusion and exclusion criteria were appropriate and did not cause a particular group of persons to bear more than their fair share of risks in participating in the trial. Persons with confirmed Ebola virus, >6 months old. Participants who, in the treating physician’s opinion, had any medical condition which could put them at unreasonably increased risk were excluded from taking part in the study. |
| **Inducements, financial benefits and financial costs** |  |
| **Independent Review** |  |
| **Resources for the research** | The funder for the trial is the Bill and Melinda Gates Foundation. The sponsor of the study is Clinical Research Management, Inc. |
| **Research staff training and protection** |  |

| **Study title** | [**Putative Investigational Therapeutics in the Treatment of Patients With Known Ebola Infection**](https://clinicaltrials.gov/ct2/show/NCT02363322?term=ebola&rank=18) |
| --- | --- |
| **Unique study number** | [5](https://clinicaltrials.gov/ct2/show/NCT02363322?term=ebola&rank=18) |
| **Study author(s)** | McConnell R et al. |
| **Ethical approval** | Yes |
| **Scientific design, conduct and validity of the study** | Randomised, safety/efficacy study, parallel assignment, open label, phase 1/2 treatment trial |
| **Favourable risk-benefit ratio** | ZMapp has been used for compassionate use on Ebola patients and results appear promising. The control group is appropriate receiving optimised standard of care alone. The potential benefit of ZMapp outweighs any known adverse effects of the drug. |
| **Protection of research participants' privacy and confidentiality** |  |
| **Informed consent process** | Inclusion criteria specifies that the ability to provide informed consent personally, or by a legally authorised representative if the patient is unable to do so is a pre-requisite to enrolling onto the study. |
| **Community considerations and collaborative partnership** |  |
| **Selection and recruitment of research participants** | Inclusion and exclusion criteria were appropriate and did not cause a particular group of persons to bear more than their fair share of risks in participating in the trial. Participants who had any medical condition which could put them at unreasonably increased risk were excluded from taking part in the study. |
| **Inducements, financial benefits and financial costs** |  |
| **Independent Review** |  |
| **Resources for the research** | The primary sponsor of the trial is the National Institute of Allergy and Infectious Diseases (NIAID). |
| **Research staff training and protection** |  |

| **Study title** | [**Efficacy of Favipiravir Against Severe Ebola Virus Disease**](https://clinicaltrials.gov/ct2/show/NCT02662855?term=ebola&rank=24) |
| --- | --- |
| **Unique study number** | [6](https://clinicaltrials.gov/ct2/show/NCT02662855?term=ebola&rank=24) |
| **Study author(s)** | Zhong W et al. |
| **Ethical approval** |  |
| **Scientific design, conduct and validity of the study** | Randomised, safety/efficacy study, parallel assignment, open label, phase 2 treatment trial |
| **Favourable risk-benefit ratio** | No previous study using Favipiravir to treat Ebola. Drug may increase chance of survival. In a previous trial versus influenza there were not major side effects but this trial is using higher doses. |
| **Protection of research participants' privacy and confidentiality** |  |
| **Informed consent process** | Inclusion criteria specifies that participant or guardian must be able and willing to provide written informed consent |
| **Community considerations and collaborative partnership** |  |
| **Selection and recruitment of research participants** | Inclusion and exclusion criteria were appropriate and did not cause a particular group of persons to bear more than their fair share of risks in participating in the trial. Inclusion criteria was persons with clinical diagnosis of Ebola virus and positive blood viral RNA detection, 13 to 75 years old. Pregnant or breast-feeding women were excluded. |
| **Inducements, financial benefits and financial costs** |  |
| **Independent Review** |  |
| **Resources for the research** | The primary sponsor of the study is the Beijing Institute of Pharmacology and Toxicology |
| **Research staff training and protection** |  |

| **Study title** | [**Clinical Trial to Evaluate the Efficacy and Safety of Convalescent Plasma for Ebola Treatment**](https://clinicaltrials.gov/ct2/show/NCT02333578?term=ebola&rank=25) |
| --- | --- |
| **Unique study number** | 7 |
| **Study author(s)** | Brown J et al. |
| **Ethical approval** | Yes |
| **Scientific design, conduct and validity of the study** | Non-randomised, efficacy study, single group assignment, open label, phase I/II pilot treatment trial |
| **Favourable risk-benefit ratio** | Yes, for both patients and plasma donors. Benefits to patient: Plasma transfusion may results in increased survival. If confirmed, this intervention could be scaled up and be beneficial for all Ebola patients and increase trust in medical profession from local community. Risks: Transmission of infection and adverse reactions relating to transfusion. Transfusion may not be acceptable for the local community. |
| **Protection of research participants' privacy and confidentiality** |  |
| **Informed consent process** | Inclusion criteria specifies that participants (or family member if potential subject unable to consent) must be able and willing to provide informed consent |
| **Community considerations and collaborative partnership** |  |
| **Selection and recruitment of research participants** | As this is a phase I/II study, children and pregnant women are excluded from taking part. Inclusion and exclusion criteria were appropriate and did not cause a particular group of persons to bear more than their fair share of risks in participating in the trial. |
| **Inducements, financial benefits and financial costs** |  |
| **Independent Review** |  |
| **Resources for the research** | The funder for the trial is the Bill and Melinda Gates Foundation. The sponsor of the trials is ClinicalRM. |
| **Research staff training and protection** |  |

| **Study title** | [**A Prospective, Open Label, Phase 1 Safety Study of Passive Immune Therapy During Acute Ebola Virus Disease Using Transfusion of INTERCEPT Plasma Prepared From Volunteer Donors Who Have Recovered From Ebola Virus Disease**](https://clinicaltrials.gov/ct2/show/NCT02295501?term=ebola&rank=37) |
| --- | --- |
| **Unique study number** | [8](https://clinicaltrials.gov/ct2/show/NCT02295501?term=ebola&rank=37) |
| **Study author(s)** | Winkler A et al. |
| **Ethical approval** | Yes |
| **Scientific design, conduct and validity of the study** | Non-randomised, safety/efficacy study, single group assignment, open label, phase 1 treatment trial |
| **Favourable risk-benefit ratio** | Favourable ratio for both patients and plasma donors. Benefits to patient: Plasma transfusion may result in increased survival. If confirmed in the United States study, this intervention could be scaled up and be beneficial for all Ebola patients. Risks: Transmission of Ebola or other infectious diseases and adverse reactions relating to transfusion. |
| **Protection of research participants' privacy and confidentiality** | Once enrolled in the study, participants are assigned a unique study number and any information with personal health identifiers such as blood samples or case report forms shared with sponsor are coded to ensure anonymity is maintained. All identifiable information kept in locked office in research institution. |
| **Informed consent process** | Both the plasma donor and recipient must provide written signed informed consent before participating in the trial. The plasma recipient's legal guardian can also provide written informed consent. Either party can withdraw from the study at any time. |
| **Community considerations and collaborative partnership** | N/A as took place in United States where there were no Ebola patients at the time so no participants to try and recruit. |
| **Selection and recruitment of research participants** | Despite being a safety, phase I study, there are no age restrictions on participants and the only exclusion criteria is food allergy to psoralens (component in active ingredient). Inclusion criteria is appropriately rigorous. |
| **Inducements, financial benefits and financial costs** | Reasonable travel compensation was offered to participants. No money was given for plasma donation. |
| **Independent Review** | FDA authorisation was required before able to proceed in addition to gaining ethical approval. |
| **Resources for the research** | The sponsor of the study was Cerus Corp. All researchers from Emory volunteered their time to help set up the study. |
| **Research staff training and protection** | There is a requirement of the sponsor that everyone is trained to minimum research standards and specific training is carried out on using the INTERCEPT plasma system. Researchers covered and employed exclusively by Cerus. |

| **Study title** | **Treating the Host Response to Ebola Virus Disease with Generic Statins and Angiotensin Receptor Blockers** |
| --- | --- |
| **Unique study number** | 9 |
| **Study author(s)** | Fedson D et al. |
| **Ethical approval** | No (it was not viewed as a clinical trial but for compassionate use) |
| **Scientific design, conduct and validity of the study** | There was no scientific "design". Patients were treated consecutively. |
| **Favourable risk-benefit ratio** | The statin/ARB combination has been shown to be more effective than single agent treatment in 40 experimental and 40 clinical studies conducted by cardiovascular investigators. Treatment with the two agents has been shown to be safe when administered to thousands of inpatients with other forms of acute critical illness. Mortality in untreated Ebola patients was 40-70%. What was there to lose by treating these patients? |
| **Protection of research participants' privacy and confidentiality** | No information available |
| **Informed consent process** | No informed consent was taken |
| **Community considerations and collaborative partnership** | No information available |
| **Selection and recruitment of research participants** | Ebola test-positive patients were treated consecutively |
| **Inducements, financial benefits and financial costs** | The drugs were provided free of charge. No payments were made to physicians who treated patents. One demanded a bribe to release data on the findings, but this was refused. |
| **Independent Review** | There was no independent review because there was no study protocol |
| **Resources for the research** | No logistical or financial support was available. The use of these agents was openly opposed by local WHO and MSF staff and by WHO staff in Geneva. One prominent Ebola scientist said this approach to treatment was "a terrible idea". |
| **Research staff training and protection** | None |

| **Study title** | [**Efficacy of Favipiravir (T-705) in patients infected with Ebola virus in Sierra Leone: a preliminary clinical trial**](http://apps.who.int/trialsearch/Trial2.aspx?TrialID=ChiCTR-OCN-15007272) |
| --- | --- |
| **Unique study number** | [10](http://apps.who.int/trialsearch/Trial2.aspx?TrialID=ChiCTR-OCN-15007272) |
| **Study author(s)** | Jiafu J et al. |
| **Ethical approval** | Yes |
| **Scientific design, conduct and validity of the study** | Non-randomised, double-blind trial. No trial phase stated. |
| **Favourable risk-benefit ratio** | No previous study using Favipiravir to treat Ebola. Drug may increase chance of survival. In a previous trial versus influenza there were not major side effects but this trial is using higher doses. |
| **Protection of research participants' privacy and confidentiality** | Blood samples of patients were destroyed after use |
| **Informed consent process** | No information on consent was stated in the inclusion criteria |
| **Community considerations and collaborative partnership** |  |
| **Selection and recruitment of research participants** | Inclusion and exclusion criteria were appropriate and did not cause a particular group of persons to bear more than their fair share of risks in participating in the trial. Pregnant women and children under 9 years old were not allowed to take part. |
| **Inducements, financial benefits and financial costs** |  |
| **Independent Review** |  |
| **Resources for the research** | The primary sponsor of the study is the Sierra Leone-China Friendship Hospital |
| **Research staff training and protection** |  |

| **Study title** | **Convalescent plasma for early Ebola virus disease in Sierra Leone** |
| --- | --- |
| **Unique study number** | 11 |
| **Study author(s)** | Semple C et al. |
| **Ethical approval** | Yes |
| **Scientific design, conduct and validity of the study** | Emergency, phase 2/3, open-label, non-randomized controlled clinical trial. Compare outcomes for standard supportive care plus convalescent plasma with supportive care plus equivalent volume of Ringer's Lactate |
| **Favourable risk-benefit ratio** | Yes, for both patients and plasma donors. Benefits to patient: Plasma transfusion may results in increased survival. If confirmed, this intervention could be scaled up and be beneficial for all Ebola patients and increase trust in medical profession from local community. Risks: Transmission of infection and adverse reactions relating to transfusion. Transfusion may not be acceptable for the local community. |
| **Protection of research participants' privacy and confidentiality** | All patients' data will be coded via a unique subject number. Any documents which allow patients to be identified will only be accessible by medical staff. Other people can only access these by signing a confidentiality agreement. Samples sent out of the country for testing will be coded. Other patients may see who is being transfused due to scarce resources at the Ebola treatment centres. |
| **Informed consent process** | No participant can be enrolled in the study until written informed consent has been obtained. Participants may withdraw from the study at any time. Measures are in place for vulnerable patients who lack capacity, children and those who are illiterate and cannot sign the consent form but still want to participate. No particular group has been disadvantaged. |
| **Community considerations and collaborative partnership** | Local communities and stakeholders were engaged with prior to the trial starting in order to ensure community acceptance. Social norms and beliefs were addressed to ensure the trial was implemented in a culturally acceptable way. Community engagement must continue following completion of the study. |
| **Selection and recruitment of research participants** | An appropriate sample size calculation was carried out. Inclusion and exclusion criteria were appropriate and did not cause a particular group of persons to bear more than their fair share of risks in participating in the trial. Pregnant women were allowed to participate in the trial. |
| **Inducements, financial benefits and financial costs** | Plasma Donors were compensated for time and travel |
| **Independent Review** | Wellcome trust and Peer Reviewed by Fred Hayden. Monitoring of the trial ensured by ClinicalRM. External monitoring scheme may not be logistically possible in which case an internal monitor may be appointed. The trial could be stopped at any point by the Data Safety Monitoring Board. |
| **Resources for the research** | Wellcome trust, Bill and Melinda Gates, Clinical RM |
| **Research staff training and protection** | Three way agreement between University of Liverpool, Clinical RM and Ministry of Health and Sanitation Republic of Sierra Leone under an Memorandum of understanding and clinical trial contract |

| **Study title** | **Rapid Assessment of Potential Interventions & Drugs for Ebola (RAPIDE) - TKM 130803 (TKM)** |
| --- | --- |
| **Unique study number** | 12 |
| **Study author(s)** | Horby P et al. |
| **Ethical approval** | Yes |
| **Scientific design, conduct and validity of the study** | Non-randomised, efficacy study, single arm, open label, treatment trial with concurrent observational study |
| **Favourable risk-benefit ratio** | The benefits of the intervention are the potential effect of TKM on reducing the current mortality rate of Ebola. In vitro studies using TKM have established evidence of efficacy against Ebola virus. The risks of the trial include: Potential adverse effects which have not been observed in safety studies on animals and healthy human volunteers. The community perceives that the intervention is no successful and so causes mistrust towards doctors. |
| **Protection of research participants' privacy and confidentiality** | Due to infection control, paper cannot be transferred outside of the wards so all data including identifiable data will be photographed and archived electronically. All identifiable data is stored only on a protected Master List and this information will only be accessed by research staff who have signed the locally appropriate data protection training. All other data will be anonymised using a unique study code for each participant. |
| **Informed consent process** | Inclusion criteria states that informed consent must be provided by the patient if >18 years old, by their representative if too unwell or by their parent or guardian if under 18 years old. Informed consent forms were submitted by and approved by the Sierra Leone Ethics and Scientific Review Committee and the Oxford Tropical Research Ethics Committee. |
| **Community considerations and collaborative partnership** | Messages will be disseminated to the community via meeting with local leaders, radio messages and posters. Ebola patients will also be offered psychological and social support. Education on the current progress of Ebola research will be provided to local health authorities. |
| **Selection and recruitment of research participants** | Inclusion criteria was appropriate and did not cause a particular group of persons to bear more than their fair share of risks in participating in the trial. All eligible participants were offered the opportunity to enrol in the study. Exclusion criteria stated participants must not have any underlying condition that could jeopardise patient or staff safety. The potential teratogenicity of TKM meant that pregnant women were excluded from participation. Similarly, TKM has never been tested in children so, initially, only those under 18 will be eligible. |
| **Inducements, financial benefits and financial costs** | Clinic and treatment costs covered by the Ebola treatment centre funding organisation for anyone attending the treatment centre regardless of study enrolment. TKM will be supplied to participants free of charge. Patients will be provided with a mobile phone to ensure follow-up and this will become the patient’s property following study completion. Reasonable travel expenses and compensation will be paid. |
| **Independent Review** | The World Health Organisation independently reviewed the protocol whilst written approval was sought from the Oxford Tropical Research Ethics Committee. |
| **Resources for the research** | Funding by the Wellcome Trust. The primary sponsor of the study is the University of Oxford. |
| **Research staff training and protection** | The University of Oxford will cover the legal liabilities of the research staff. The trial will initially take place at hospitals managed by MSF where all health care personnel must meet minimum training standards. |

| **Study title** | [**Treatment of Ebola virus disease with TCM (traditional Chinese medicine): a prospective clinical study**](http://apps.who.int/trialsearch/Trial2.aspx?TrialID=ChiCTR-OON-14005558) |
| --- | --- |
| **Unique study number** | [13](http://apps.who.int/trialsearch/Trial2.aspx?TrialID=ChiCTR-OON-14005558) |
| **Study author(s)** | Guo Y et al. |
| **Ethical approval** | Yes |
| **Scientific design, conduct and validity of the study** | Observational, case series assessing efficacy of Traditional Chinese Medicine versus symptomatic treatments using western medicine. No trial phase stated. |
| **Favourable risk-benefit ratio** |  |
| **Protection of research participants' privacy and confidentiality** |  |
| **Informed consent process** | Inclusion criteria states that patients must enrol voluntarily and sign the informed consent form. |
| **Community considerations and collaborative partnership** |  |
| **Selection and recruitment of research participants** | Inclusion criteria was appropriate and included all persons with clinical diagnosis of Ebola virus who were aged 18 to 65 years old. Exclusion criteria stated that pregnant women could not take part in the trial. Those with seriously abnormal liver, heart and kidney functions were also excluded as were those with a mental disorder, another infectious disease, poor adherence to Traditional Chinese Medicine or if death occurred within 24 hours of admission. |
| **Inducements, financial benefits and financial costs** |  |
| **Independent Review** |  |
| **Resources for the research** | The primary sponsor of the study is the 302 Military Hospital of China |
| **Research staff training and protection** |  |

| **Study title** | [**Rapid Assessment of Potential Interventions & Drugs for Ebola (RAPIDE) - BCV**](http://apps.who.int/trialsearch/Trial2.aspx?TrialID=PACTR201411000939962) |
| --- | --- |
| **Unique study number** | [14](http://apps.who.int/trialsearch/Trial2.aspx?TrialID=PACTR201411000939962) |
| **Study author(s)** | Horby P et al. |
| **Ethical approval** | Yes |
| **Scientific design, conduct and validity of the study** | Non-randomised, efficacy study, single arm, open label, treatment trial historical controls used |
| **Favourable risk-benefit ratio** | Brincidofovir has demonstrated antiviral activity versus Ebola in vitro in both human and animal models. However, there are a number of risks from brincidofovir treatment including gastro-intestinal side-effects, potential carcinogenic effects and as well as the risk of the community distrusting doctors if the intervention does not work. These risks are considered to be outweighed by the potential benefit of the drug on the case fatality rate of 70.8% of the Ebola virus. Babies under two months and pregnant women have been excluded due to the potential and unknown embryo/foetal effects of the drug. |
| **Protection of research participants' privacy and confidentiality** | Due to infection control, paper cannot be transferred outside of the wards so all data including identifiable data will be photographed and archived electronically. All identifiable data is stored only on a protected Master List and this information will only be accessed by research staff who have signed the locally appropriate data protection training. All other data will be anonymised using a unique study code for each participant. |
| **Informed consent process** | Participants aged 18 or older must provide fully informed consent or the provision of consent from the representative of a patient who is too unwell to consent. For those aged under 18, consent from parent or guardian is required. Participants can withdraw at any time and they will then receive the best available care minus the intervention. |
| **Community considerations and collaborative partnership** | MSF will be responsible for disseminating messages to the community via meeting with local leaders, radio messages and posters. Ebola patients will also be offered psychological and social support. Education on the current progress of Ebola research will be provided to local health authorities. |
| **Selection and recruitment of research participants** | Inclusion criteria was appropriate and did not cause a particular group of persons to bear more than their fair share of risks in participating in the trial. All eligible participants were offered the opportunity to enrol in the study. Exclusion criteria stated participants must not have any underlying condition that could jeopardise patient or staff safety. Pregnant women were excluded. |
| **Inducements, financial benefits and financial costs** | Clinic and treatment costs covered by MSF for anyone attending the treatment centre regardless of study enrolment. Brincidofovir will be supplied to participants free of charge. Patients will be provided with a mobile phone to ensure follow-up and this will become the patient’s property following study completion. Reasonable travel expenses and compensation will be paid. |
| **Independent Review** | The World Health Organisation independently reviewed the protocol whilst written approval was sought from the Oxford Tropical Research Ethics Committee. |
| **Resources for the research** | Funding by the Wellcome Trust. The primary sponsor of the study is the University of Oxford. |
| **Research staff training and protection** | The University of Oxford will cover the legal liabilities of the research staff. The trial will initially take place at hospitals managed by MSF where all health care personnel must meet minimum training standards. |

| **Study title** | [**Investigation on the efficacy and safety of favipiravir in patients who are infected or strongly suspected of being infected with Ebola virus**](http://apps.who.int/trialsearch/Trial2.aspx?TrialID=JPRN-UMIN000016101) |
| --- | --- |
| **Unique study number** | 15 |
| **Study author(s)** | Kato Y et al. |
| **Ethical approval** | Yes |
| **Scientific design, conduct and validity of the study** | Non-randomised, Uncontrolled, Efficacy/Safety study, Single-arm, open-label trial |
| **Favourable risk-benefit ratio** | No previous study using Favipiravir to treat Ebola. Drug may increase chance of survival. In a previous trial versus influenza there were not major side effects but this trial is using higher doses. |
| **Protection of research participants' privacy and confidentiality** | The protocol strictly follows the Declaration of Helsinki and the national ethics guidance on human medical research. The IRB approved the study. Collected biological materials will be stored at the National Institute of Infectious Diseases according to national law. |
| **Informed consent process** | Patients aged 20 years or older must submit written informed consent. Proxy consent is acceptable if patient is incompetent to consent. For junior-high and high-school students, both patient and parent/guardian consent is required. For primary school children or younger, written consent by their parent/guardian only is required. |
| **Community considerations and collaborative partnership** | The research protocol was integrated by the Japanese government into the national public health response against the Ebola epidemic in West Africa. |
| **Selection and recruitment of research participants** | Inclusion and exclusion criteria were appropriate and did not cause a particular group of persons to bear more than their fair share of risks in participating in the trial. Any patient who was considered as inadequate for the study by the Case Management Committee was excluded. |
| **Inducements, financial benefits and financial costs** | Medical care will be provided free of charge in accordance with Japanese law. No monetary rewards will be given to participants in the study. |
| **Independent Review** | The protocol strictly follows the Declaration of Helsinki and the national ethics guidance on human medical research. The IRB approved the study. |
| **Resources for the research** | The sponsor for the study is the National Centre for Global Health and Medicine (Japan). Funding is from the Ministry of Health, Labour and Welfare, Japan. |
| **Research staff training and protection** | All researcher received training on infection prevention and control. |

| **Study title** | **A pilot study to evaluate the safety and efficacy of interferon beta-1a for the treatment of patients with Ebola virus** |
| --- | --- |
| **Unique study number** | 16 |
| **Study author(s)** | Fish E et al. |
| **Ethical approval** | Yes |
| **Scientific design, conduct and validity of the study** | Non-randomised pilot phase I/II intervention trial assessing safety and efficacy of IFN beta-1a versus usual supportive care only. |
| **Favourable risk-benefit ratio** | Risks and benefits have been listed in the trial protocol. Procedures such as aseptic non-touch technique will be enforced to minimise risk. The drug has not been administered to patient with Ebola before; however, reviews have shown IFN-beta-1a to have a favourable safety profile. This, coupled with the potential benefit to patients of increased recovery from Ebola give a favourable risk-benefit ratio. |
| **Protection of research participants' privacy and confidentiality** | Participants can withdraw from the study at any time without risking their future care. |
| **Informed consent process** | Written informed consent will be taken from each participant before enrolling in the study. |
| **Community considerations and collaborative partnership** | Dr. Mandy Kader Konde is a Guinean national and has a research centre and clinic in the area where the trial is taking place so has a working relationship with the community. No Caucasian involved to maintain culturally sensitivity but still lots of fears about injections as people think they were being injected with the virus. |
| **Selection and recruitment of research participants** | There is no sample size as the study is to prove feasibility. Inclusion and exclusion criteria are appropriate and evidence-led. Pregnant women are not allowed to enrol in the trial. Participants will be recruited from one hospital in Guinea if there are suspected or known to have Ebola. |
| **Inducements, financial benefits and financial costs** | None as not enough funding received to offer compensation or expenses payments to participants. |
| **Independent Review** | Shared protocol with WHO and company that provided the drug. |
| **Resources for the research** | Reported incidents to Guinean Government directly under their jurisdiction. This information was provided by Dr. Mandy Kader Konde. Living costs of research team paid via $80000 grant from Canadian Institutes of Health Research. |
| **Research staff training and protection** | Twelve Guineans were trained can now run a clinical trial to European standards including PPI equipment, consent forms etc. If staff infected would have made sure |
