# Supplementary material for: A Systematic Review of Ebola Treatment Trials to Assess the Extent to Which They Adhere to Ethical Guidelines
Source: PLoS One. 2017 Jan 17;12(1):e0168975. doi: 10.1371/journal.pone.0168975 (PMC5240928; doi:10.1371/journal.pone.0168975)
Supplement: S6 Appendix — (DOCX) [file pone.0168975.s006.docx]

**S6 Appendix. Detailed outline of the three ethical frameworks by the WHO, MSF and Emanuel et al.**

**WHO framework**

Scientific design and conduct of the study

- Valid scientific methods
- Examine ethical implications of the chosen research design
- Assess how study conducted, qualifications of researchers, adequacy of provisions for monitoring/auditing and adequacy of study site

Risks and potential benefits

- Risks minimised and reasonable in relation to potential benefits of study
- Nature of risks differ according to type of research and can be physical, psycho, financial or social
- Harm can occur at individual, family or population level

Selection of study population and recruitment of research participants

- No group of persons bears more than fair share of burdens of participation in research
- No group deprived of its fair share of benefits- direct or indirect (new knowledge that research yields)
- Whether the population that will beat h risks of participating in the research are likely to benefit from the knowledge derived from the research
- Recruitment strategy should be balanced and objectively describe the purpose of the research, the risks and potential benefits of participating in the research and other relevant details

Inducements, financial benefits and financial costs

- Reimburse individuals for any costs associated with participation in research (transport, child care, lost wages) and maybe to compensate for time
- Payments not so large/ not free medical care as to induce pt. to consent against their better judgement or to compromise their understanding of the research

Protection of research participants’ privacy and confidentiality

- Invasion of privacy or breaches of confidentiality are disrespectful to pt.
- Can lead to loss of control or embarrassment or social stigma, rejection by families, loss of employment or housing
- Should be precautions to safeguard pt. privacy and confidentiality

Informed consent process

- Respect for persons
- Competent individuals choose freely whether to participate in research and make decision based on adequate understanding of what the research entails
- Children or adults who lack mental capacity to provide informed consent made by authorised surrogate decision maker
- Process through which informed consent made and info provided
- Waive requirement for informed consent only when consistent with international guidelines and national standards
- Informed consent by participant alone does not mean that research is ethically acceptable

Community considerations

- Impact of research on communities where research occurs and to whom findings can be linked as well as individual
- Respect and protect communities to minimise any negative effects such as stigma or draining of local capacity
- Promote positive effects on communities including related to health effects or capacity development
- Researchers should actively engage with communities about the design and conduct of research (including informed consent process)
- Researchers should be sensitive to and respect communities’ cultural, traditional and religious practices

**Emanuel et al. framework**

Collaborative partnership

- Which community representatives will be partners, involved in helping to plan and conduct the research, disseminate the results and use the results to improve health?
- How will responsibility be shared with these partners for planning and conducting the research, disseminating the results and using the results to improve health?
- How will respect for the community’s values, circumstances, culture, social practices and so forth, be demonstrated?
- How will fair benefits for the community from the conduct and results of the research be assured?
- How will the tangible benefits of the research, such as authorship credit and intellectual property rights, be distributed to ensure fairness?

Social value

- Who will benefit from the conduct and results of research?
- What is the potential value of the research for each of the prospective beneficiaries?
- How will the social value of the research be enhanced?
- How can the adverse impacts, if any, of conducting the research be minimised?

Scientific validity

- Do the scientific and statistical design and methods satisfy generally accepted standards and achieve the objectives of the study? If not, is their clear justification for the deviations?
- Will the research results be interpretable and useful in the context of the health problem?
- Does the study design ensure participants health-care services they are entitled to? If not, are the methodically compelling reasons and are participants protected from serious harm?
- Is the research design practically feasible given the social, political, economic and cultural environment?

Fair participant selection

- Is the research population selected to ensure that the research complies with scientific norms and will generate valid and reliable date?
- Is the research population selected to minimise risks to the participants?
- Are the individual research participants selected to maximise social value and enhance the possibility of benefits to the participants?
- Are the participants vulnerable based on age, clinical status, social marginalisation, economic deprivation, and so forth? If so, what safeguards are included to protect the participants?

Favourable risk-benefit ratio

- Are the potential physical, psychological, social and economic risks of the research for the individual participants delineated and their probability and magnitude quantified to the extent possible given the available data?
- Are the potential physical, psychological, social and economic benefits of the research for the individual participants delineated and their probability and magnitude quantified to the extent possible given the available data?
- When compared, do the potential benefits to the individual outweigh the risks? If not, does the knowledge gained from the study for society justify the net risks to the individual participants?

Independent review

- Are the procedure for independent review established by law and regulation being properly followed?
- Is the review body both independent and competent?
- Is the review process transparent and are reasons given for the review committee’s decisions?
- Are multiple reviews minimised and reconciled if they conflict?

Informed consent

- Are recruitment procedures and incentives consistent with cultural, political and social practices of the potential participants and their community?
- Are disclosure forms and verbal disclosure procedures sensitive to participants’ culture, language and context?
- Is the information presented to participants complete, accurate and not overwhelming?
- Are there appropriate plans in place for obtaining permission from legally authorised representatives for individuals unable to consent for themselves?
- Are supplementary consents or permissions, for example, from spouses or community leaders, obtained? If so, are there ways to ensure that the individual participant can still decide whether to participate independent of the spouse or community leader?
- Are the mechanisms to symbolize consent consistent with the participants’ culture and context?
- How will the individual participants be made aware of their right to refuse to participate and are they actually be free to refuse?

Respect for participants

- How will the health and well-being of participants be monitored to minimise harms? Are the criteria for changing doses or procedures for stopping the study for the health of participants adequate?
- How will the confidentiality procedures actually be implemented?
- How will it be ensured that participants who want to withdraw can withdraw without penalty?
- How will the results of the research be disseminated?
- What are the plans for care of the participants after the research is completed?

**MSF framework**

Research Question and Methodology

1. What is the research question? Why is it important?

- Research question should be central element in any protocol
- Where more than one question, they should be presented in logical order
- Why is the RQ scientifically important? What knowledge gap will it fill?
- Why is the RQ important to the community affect?
- If other alternative RQ’s are possible, why was this particular question selected?
- What potential harms might arise if research is not conducted?

1. How is the methodology and proposed analysis appropriate given the research question(s)?

- Proposed method and analysis will not only allow researchers to answer the question that they have set, but that it is the best way to do so
- How will the research design and analysis provide the best means of answering the proposed question (e.g. sample size and method, selection of study population etc.)?
- What scientific/methodology review has been obtained prior to submission for ethical review?
- How have ethical considerations shaped the proposed methodology? For example, what justification exists for any standard of care in the proposed research?

1. What is the context in which the research will be conducted? How has this influenced the research design?

- Protocol must include details about existing and planned community engagement and collaborative partnerships and how they have influences or shaped the proposed research
- How have the community’s views about their needs and research priorities been taken into account? What is the researchers’ strategy to engage the community as part of the research process?
- What collaborative research partnerships or agreements exist in relation to this project? What engagement has occurred with local or national health authorities?
- To what extent can partnerships be structured in a fair and equitable manner?
- How will the researchers enhance local research capacity with this project?
- Has research ethics review been obtained by all appropriate ethics review boards at the local/regional/national level?

1. Are there any other parties involved in the research? What potential interests of these parties might conflict with MSF’s mission and values?

- Who may benefit directly and indirectly from the research?
- Where other parties (e.g. companies) benefit from the research, how will the interests of participants, community and MSF be protected?
- What are the potential benefits relating to spin-off interests or intellectual property etc.? How will they be apportioned?

1. Are all relevant resources for the research secured?

- What is the budget for the research? Is it secured?
- What additional infrastructure is required? Is it secured?
- What possible changes might occur in the field? What plans are in place to respond to such alterations?
- Is there an operational commitment for the expected time of the study?

1. Have the research staff the relevant training and protection?

- Have the research staff the required expertise to carry out the research?
- What training has been conducted with the research staff, or how will this be provided?
- What risks of harm might researchers be exposed to? How can this be minimised?
- Have any of the research staff double allegiances (being both carer and researcher)? How will potential conflicts of interest be avoided?

Respecting and Protecting Research Participants and Communities

1. What are the anticipated harms and benefits?

- Considering all relevant harms and benefits is an essential part of assessing whether a proposed piece of research is ethical. As MSF works mostly with populations at risk, there are multiple opportunities for considerable harm
- Given the best available evidence and any relevant experience, what are the anticipated harms and benefits of the research? How likely and how significant are any harms and benefits to research participants?
- What are the potential wider social harms and benefits to communities?
- What protections will be put in place to avoid or mitigate anticipated harms?
- Benefits and burdens of research may be unequally distributed between sub-groups. How are harms and benefits distributed between participants and communities? Have researchers ensured that any proposed inclusion/exclusions criteria are fair?
- What is he process to monitor unknown harms/ new information arising in the study? Will a data and safety monitoring committee be needed?

1. What are your plans for obtaining consent?

- A requirement to inform participants is often seen as being an important way to show respect and promote patient autonomy and welfare
- What information ought to be provided? This will usually include the following elements: the reasons for doing the research, details about who is doing the research, why the potential participant is being asked to be involved, details about what any intervention might involve and any on-going commitments of participant, details about anticipated risks and benefits, the fact that participants are free to refuse or withdraw, that any findings will be communicated back to the participants etc. The information given should be proportionate to any risks, but this does not mean that the higher the risk, the more information ought to be provided. Sometimes, calling attention clearly to a common or significant particular risk is more important than listing every possible remote risk.
- Providing information does not guarantee it has been understood. How can information be provided at an appropriate linguistic level, without jargon or technical terms, and appropriate to the local language and culture?
- Should information be provided in oral and /or written form?
- How will the consent process be conducted? You may want to consider issues such as: who will consent, where they will do so (is the place appropriate to allow a confidential discussion), will a witness to the consent be required, how much time will be offered to consider whether to be involved? Prior engagement with communities can be a useful way to ensure that the consent process meets local expectation and sensitivities. How will the act of consent be recorded (e.g. signed and witnessed document, thumb print etc.)?
- Alternative or additional consent procedures may need to be developed where potential participants are minors, minor parents, or suffering from short or long-term incapacities etc.
- It should not be assumed that a long and complicated information sheet is always necessary and in exceptional cases it may be justifiable not to seek informed consent. Where researchers believe this is appropriate, they should be careful to provide reasons for this in the protocol.

1. How do you plan to protect confidentiality?

- Data will include all information (medical and non-medical) about or derived from participants.
- What data securities are in place?
- Where will data be gathered and stored? Who will have access to it? Where will it go?
- Will it be anonymised or coded? Will it be linked, or could it be linked, to other data sets? If so, are adequate protections in place?
- Will data be placed in the public domain (in line with the MSF data sharing policy)? How will confidentiality be protected?

1. How do you plan to access, store and distribute any collected biological material?
2. Will biological material be collected, retained, sored, exported or destroyed? If so, how will this be done? If collected for one purpose, could it be used for other purposes?
3. Is the relevant consent obtained?
4. Where transfer of material is planned what nationals or international regulations are relevant? Have the necessary authorisation been sought? Is there a material transfer agreement in place? If so, what does this say?

Implications and Implementation of the Research Findings

1. What will happen when the research is either stopped or is complete?
2. Good planning for a project will consider how it will end

- Under what conditions would you consider stopping the project earlier than planned?
- What will happen to investments in infrastructure, human and other resources, when the research is complete or ends early?

1. How will the findings be disseminated?

- How will the results be disseminated? Through publication? Where? Will they be available through open access or on the MSF website?
- How will MSF communicate the results of the research directly to the community/participants involved?
- What is the plan for dissemination if the research findings are negative?

1. How will the findings be implemented?

- If will not be possible, before results are known, to establish all the details about implementation. However, it is often possible to think about such issues in advance
- What is MSF’s obligation to the research participants?
- What is MSF’s obligation to others in the same situation elsewhere?
- How will MSF fulfil any post-research obligations entailed by the results of the research?
- Is there an (advocacy) plan in place to assure access to benefits of the study results if applicable? This is particularly important where individuals and communities are unable to access and intervention for some reasons (e.g. it is too expensive)
